# Insertion of a Divergent GAF-like Domain Defines a Novel Family of YcgR Homologues That Bind c-di-GMP in *Leptospirales*

**DOI:** 10.1021/acsomega.4c09917

**Published:** 2025-01-21

**Authors:** Aline Biazola Visnardi, Rodolfo Alvarenga Ribeiro, Anacleto Silva de Souza, Tania Geraldine Churasacari Vinces, Edgar E. Llontop, Anielle Salviano de Almeida Ferrari, Pedro Antônio França Henrique, Daniela Valdivieso, Daniel Enrique Sánchez-Limache, Gabriela Roberto Silva, Eduardo Pereira Soares, Thomas Wittmann Cezar Santos, Chuck Shaker Farah, Rogerio Corte Sassonia, Roberto K. Salinas, Cristiane Rodrigues Guzzo, Robson Francisco de Souza

**Affiliations:** † Department of Microbiology, Institute of Biomedical Sciences, University of São Paulo, São Paulo 05508-060, Brazil; ‡ Department of Parasitology, Institute of Biomedical Sciences, University of São Paulo, São Paulo 05508-060, Brazil; § Department of Biochemistry, Institute of Chemistry, University of São Paulo, São Paulo 05508-060, Brazil; ∥ Federal University of São Paulo, Department of Chemistry, São Paulo 05508-060, Brazil; ⊥ Graduate Program in Bioinformatics, University of São Paulo, São Paulo 05508-060, Brazil

## Abstract

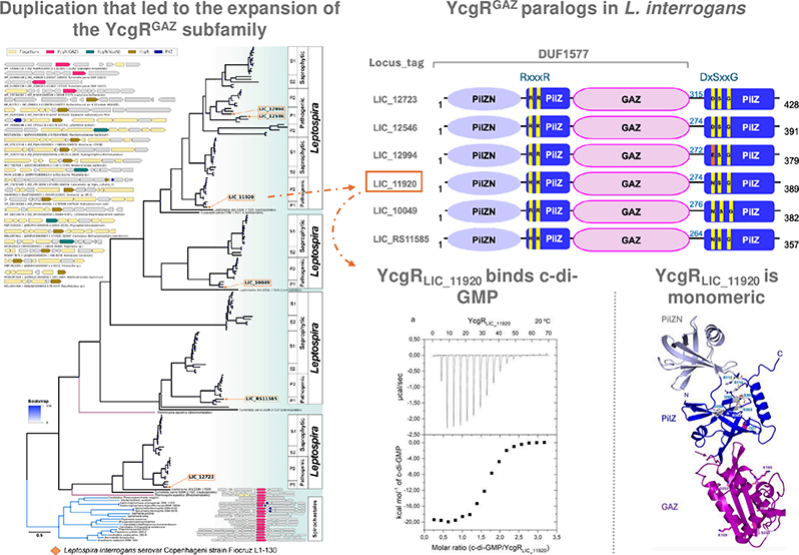

The Leptospiraceae family, which includes the genera *Leptospira, Leptonema*, and *Turneriella*, is an ecologically diverse group that includes saprophytic strains from soil and water as well as important pathogenic strains. Adaptation to these multiple environments relies strongly on signal transduction to adjust their morphology, motility, and metabolism to the changing environmental conditions. Members of the genus *Leptospira* distinguish themselves among spirochetes for having an elevated number of signal transduction genes. In this study, we describe a novel signal transduction protein that has gained multiple paralogues in the Leptospiraceae. These proteins are members of the YcgR/DgrA/MotI family, whose orthologs in several bacterial lineages have been shown to regulate the flagellar motor upon binding to c-di-GMP through their N-terminal PilZ domain. Unlike previously described versions of YcgR, the spirochetal proteins are characterized by the insertion of a divergent GAF domain within their N-terminal PilZ domain. We show that one member of this protein family from *Leptospira interrogans* is still a monomeric c-di-GMP binding protein and that these novel YcgR-like proteins have mostly replaced other members of the YcgR family in Leptospiraceae. Marked divergence among the paralogs suggests this family’s expansion was accompanied by neofunctionalization, with the likely emergence of novel interactions in the signal transduction network controlling the flagellum rotor and other processes affected by changes in levels of c-di-GMP.

## Introduction

The family *Leptospiraceae*, a division within the order Spirochaetales, encompasses the genera *Leptospira*, *Leptonema*, and *Turneriella*. Species in the genus *Leptospira* can thrive in diverse ecological niches and life styles, including saprophytic and pathogenic species. Pathogenic *Leptospira* are responsible for leptospirosis, a significant zoonotic disease of mammals in tropical regions.^1^ Among the more than 300 serovars described for the species *Leptospira interrogans*, the serovar Copenhageni stands out as the main etiological pathogen for human leptospirosis, a neglected tropical disease. Ongoing climate change and increasing average global temperatures may turn leptospirosis into a worldwide public health issue. The clinical symptoms of this disease may escalate to a severe condition, leading to jaundice, renal and hepatic failure, meningitis, hypotension, bleeding, multiple organ failure, pulmonary hemorrhages, and, in some instances, death.^2,3^ The diversity of environments in which *Leptospira* can thrive, ranging from soil and water to various mammalian organs and the bloodstream, underscores the need for distinct signaling pathways to sense external surroundings, modulating bacterial biochemistry, physiology, and behavior.

A key molecule in bacterial signal transduction is the second messenger bis(3′ → 5′) cyclic dimeric guanosine monophosphate (c-di-GMP), a small molecule synthesized from GTP by diguanylate cyclases (DGCs) and hydrolyzed by phosphodiesterases (PDEs).^4−8^ This universal eubacterial messenger orchestrates lifestyle transitions from individual mobile cells in planktonic growth to multicellular communities forming biofilm structures. C-di-GMP also regulates other biological processes such as secondary metabolite production; many aspects of environmental adaptation, transcription, DNA repair, protein and polysaccharide secretion; and virulence factor expression. Furthermore, c-di-GMP signaling plays a pivotal role during infection by human pathogens such as *Pseudomonas aeruginosa*, *Clostridium difficile*, *Salmonella typhimurium*, *Vibrio cholerae*, *B. burgdorferi*, and *Y. pestis*. Similar roles in pathogenicity have also been described for other hosts, such as plant pathogens *X. campestris* and *X. citri*.

To carry out its cellular functions, c-di-GMP interacts with a diverse range of effector molecules, such as riboswitches and c-di-GMP binding domains present in transcription factors, enzymes, and other targets. Among these, the PilZ domain was the first protein domain to be identified as a c-di-GMP receptor and remains one of the most extensively studied and pivotal receptors. Different PilZ-containing proteins exhibit varying affinities for c-di-GMP.^9^ Canonical PilZ domains bind to c-di-GMP by the conserved RxxxR and [D/N]XSXXG motifs,^9−11^ and notable examples include the Alg44 protein from *P. aeruginosa*, which is involved in alginate synthesis, DgrA from *C. crescentus* that affects motility,^12^ and BcsA from *G. xylinus* that regulates cellulose synthesis.^13−15^ Other PilZ proteins do not bind c-di-GMP but still regulate their targets through protein–protein interactions, such as *P. aeruginosa*’s PilZ protein (NCBI’s locus tag PA2960) and *X. citri*’s XAC1133 protein. These two proteins regulate type IV pili formation through protein interactions with PilB and FimX.^13,16,17^ Evolution of PilZ-containing proteins in all bacterial lineages was marked by some important evolutionary events, such as (i) fusions to other genes, yielding functionally diverse multidomain proteins; (ii) insertion of ∼200-bp fragments, leading to the emergence of tetramer-forming PilZ proteins; and (iii) tandem duplication of pilZ genes, giving rise to PilZ dimers and the YcgR-like proteins.^18^

Beyond c-di-GMP, bacteria produce other cyclic dinucleotides, such as cyclic-bis(3′ → 5′)-dimeric AMP (c-di-AMP)^19^ and cyclic guanosine (3′ → 5′) monophosphate-adenosine (3′ → 5′) monophosphate (cGAMP). The bacterial kingdom also employs diverse cyclic oligonucleotides, including dipurines, hybrids of purine and pyrimidines, and cyclic trinucleotides^20^ as sensing molecules in a range of signaling mechanisms, involving protein and RNA receptors. For example, c-di-AMP targets proteins with RCK domains, the universal stress protein (USP) domain, and PstA proteins.^21^ Conversely, the understanding of bacterial cGAMP receptors remains limited, with one example being the CapV phospholipase from *V. cholerae*.^22^

The role of c-di-GMP in bacteria allows for rapid responses through direct protein regulation or slower responses by modulation of gene expression. Furthermore, this molecule is key to regulating both flagellar gene expression and motor function. The c-di-GMP regulatory role in bacterial motility is exemplified by the YcgR receptor, which acts as a molecular clutch or brake in flagellar rotation and is observed in various bacterial species. Torque regulation occurs through the interaction between the rotor and the stator (MotA_5_-MotB_2_).^23^ The YcgR-c-di-GMP complex binds to the stator protein MotA through its PilZ domain at the MotA-FliG interface.^24^ Simultaneously, it interacts with various motor proteins through its PilZN domain, with FliG being the preferred target, thereby regulating bacterial swimming.^24^ These regulatory interactions have been validated for the YcgR protein from *E. coli*(25) and are expected to play a similar role in YcgR homologues from other organisms, such as MotI (DgrA) from *B. subtilis*(25,26) and FlgZ from *P. aeruginosa*.

On the other hand, the mechanisms of regulation of the flagellum rotor by c-di-GMP in members of the family Leptospiraceae are largely unknown. The flagellum in this group of bacteria diverges from other bacteria, as they are characterized by the presence of periplasmic flagella, with the flagellin filament extending within the periplasm from the two poles of the cell. Spirochetes and externally flagellated species share fundamental motor components, including a rotor and a dozen stator units.^27^ However, cryo-electron tomography revealed that the rotor ring in the spirochete motor is larger than external flagellar motors, with rotor radius of 31 nm for *B. burgdorferi*, but only 20 nm for *S. enterica*, 22 nm for *V. fischeri*, and 27 nm for *C. jejuni*. This larger rotor ring allows for more stators to surround the rotor, suggesting a potentially more intricate mechanism of torque regulation. The so-called “P-collar”, a large structure observed only in the flagella of spirochetes such as *T. primitia*, *T. pallidum*, *B. burgdorferi*, *L. interrogans*, and *L. biflexa*,^28^ could also interact with the stator and/or play a role in stator assembly and function.^29^ The spirochetal motor also stands out for its ability to generate higher torque, as supported by motility measurements in *Leptospira spp*. that reported a stall torque of approximately 4000 pN nm,^30^ whereas *E. coli’*s stall torque is around 2000 pN nm.

In Leptospiraceae, rotation of the supercoiled flagella and its interaction with the cell body bends the cell ends into well-defined hook-like or spiral morphologies, depending on the direction of rotation of the flagellum.^31^ The advancing part of motile cells is spiral-shaped, while the back end adopts a hook-like morphology. When both ends have the same morphology, the cells are nonmotile;^32^ therefore, careful coordination of the activity of the two distal flagellar motors is required to ensure cell motility and shape. Although chemotactic regulators of flagellar movement, such as the flagellum collar component FliY, have already been studied in *Leptospira*, the connection between c-di-GMP signaling and flagellar torque has never been studied in these organisms, and c-di-GMP signaling pathways in *Leptospira* are still poorly characterized.^4^

In this study, we present a previously unidentified subfamily of YcgR orthologs, termed YcgR^GAZ^. The YcgR^GAZ^ proteins are distinguished by a unique protein architecture featuring a divergent GAF domain integrated into the PilZ domain. Notably, this insertion does not hinder the ability of the PilZ domain of YcgR^GAZ^ to bind c-di-GMP and may provide novel regulatory sites for this protein. We provide experimental evidence that YcgR_LIC_11920_, a YcgR^GAZ^ member in *Leptospira interrogans*, binds c-di-GMP. We also found that *L. interrogans* serovar Copenhageni has six copies of YcgR^GAZ^, while most organisms, including several spirochaetes, have only a few members of the YcgR gene family. The expansion of the YcgR^GAZ^ family in *Leptospira* may have rewired this organism’s c-di-GMP regulatory network, thus offering novel valuable targets for the development of novel antibiotics against this pathogen, and could also shed new light on the diverse biological roles played by this ubiquitous second messenger.

## Results

### *Leptopira interrogans* Encodes Many Noncanonical YcgR Proteins

In order to understand the c-di-GMP signaling network in *Leptospira interrogans* serovar Copenhageni Fiocruz L1–130, the primary causative agent of human leptospirosis in Brazil,^33,34^ we searched for proteins containing PilZ domains in the complete genome of this strain. We began by retrieving all *Leptospira interrogans* serovar Copenhageni str. Fiocruz L1–10 and *Leptospira interrogans* serogroup Icterohemaemorrhagiae serovar Lai proteins annotated with the “PilZ” keyword in the Pfam and Uniprot databases. This analysis recovered the following genes: LIC_10128 (LA_01420), LIC_12491 (LA_1204), LIC_12723 (LA_0920), and LIC_RS11585 (LA_1489). Two incorrectly annotated genes, LIC_11628 (LA_2311) and LIC_11993 (LA_1910), were also recovered but later discarded based on structure similarity searches using their AlphaFold predicted structures. Next, we searched for paralogs of LIC_12723 both using the KEGG (Kyoto Encyclopedia of Genes) web interface and by running PSI-BLAST searches initiated with LIC_12723’s amino acid sequence against the proteins of *L. interrogans* Copenhageni serovar Fiocruz L1–130 (*L. interrogans* L1–130). These searches recovered four new PilZ containing proteins: LIC_12546, LIC_12994, LIC_11920, and LIC_10049. Interestingly, the genes LIC_20173 and LIC_11447 were also recovered by searching for PilZ annotations in other *Leptospira* genomes and searching for orthologs in *L. interrogans* L1–130 genomes.

**Figure 1:**
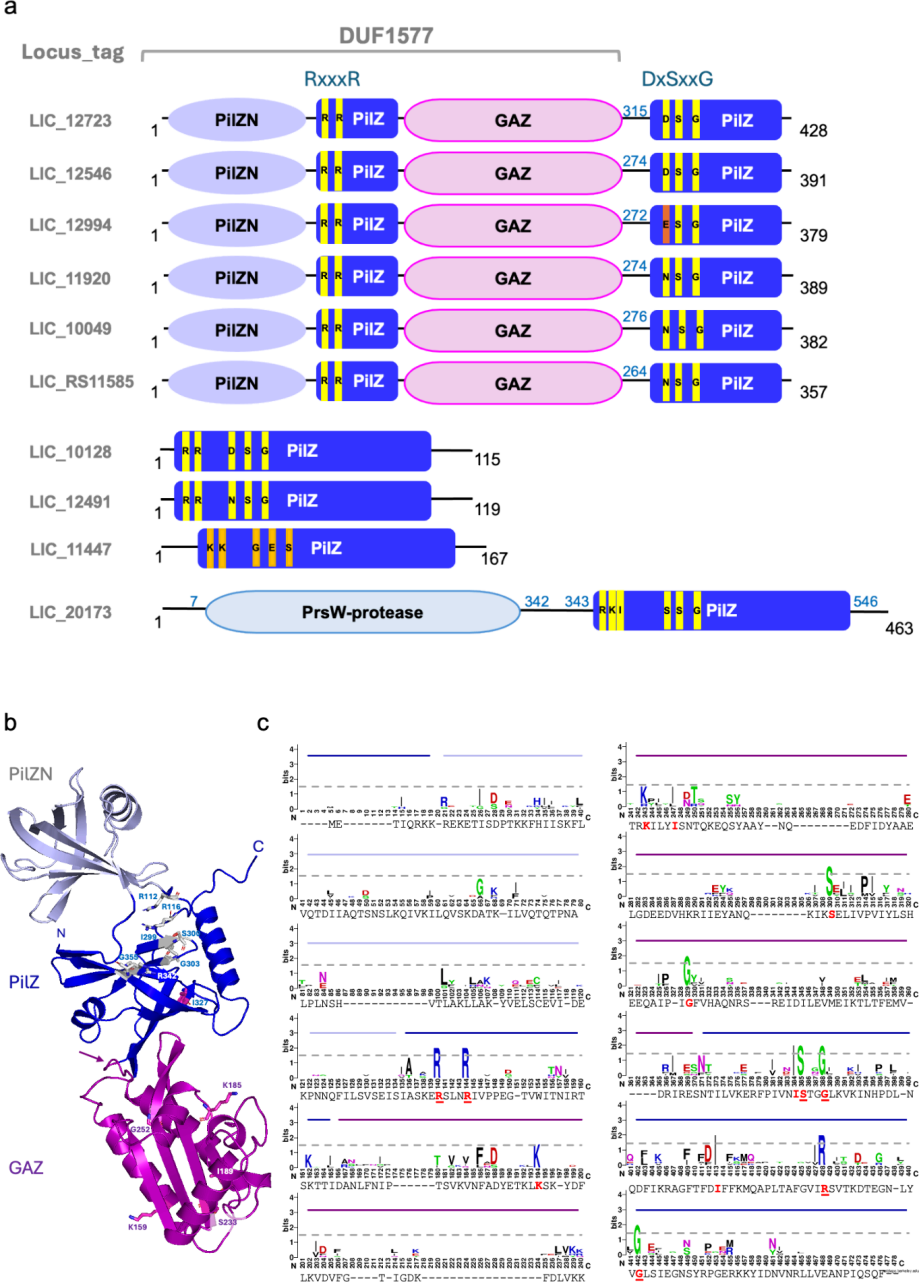
Representation of *L. interrogans* genes containing the PilZ domain. (a) Domain architecture of *L. interrogans* proteins containing the PilZ domain. The canonical residues RxxxR and DxSxxG are highlighted in yellow. LIC_20173, whose PilZ domain is not recognized by the UNIPROT and NCBI databases, is the only protein containing a PilZ domain in *L. interrogans* Copenhageni Fiocruz L1–130 that is fused to an intramembrane metalloprotease domain of the PrsW family. (b) AlphaFold2 model of the protein encoded by NCBI’s locus tag LIC_11920, showing the PilZN domain (colored in gray), GAZ (colored in purple), and PilZ (colored in blue). Conserved residues among the 6 paralogues of *L. interrogans*, shown in panel a, are shown as sticks. (c) WebLogo representation of the amino acid residue conservation pattern for the 6 paralogs of LIC_11920 from *L. interrogans* shown in panel a. The dotted gray line marks the conservation cutoff for the residues. In panel c, the upper gray line marks the residues of the PilZN domain; the blue line marks the PilZ domain; and the purple line identifies residues of the GAZ domain. The amino acid sequence below the blog corresponds to the LIC_11920 protein, with residues involved in c-di-GMP binding highlighted in red.

Protein domain architectures based on the Pfam database (Figure 1a) revealed that six of these proteins match the DUF1577 model in their N-terminal regions. Further investigation, employing tools such as Phyre2 and jackhmmer, revealed that the region that aligns to the DUF1577 model comprises a fusion of a PilZN (previously described as YcgR_N) and a GAF-like domain, forming a distinctive architecture (Figure 1). The most common domain architectures among proteins containing PilZN domains are those where an N-terminal PilZN is followed by a C-terminal PilZ. Examples of this architecture include *Escherichia coli*’s YcgR, *B. subtilis* MotI, and *P. aeruginosa*’s FlgZ. However, all *L. interrogans* L1–130 homologues of YcgR we found contained the GAF-like insertion. We therefore refer to this divergent GAF as the GAZ domain, for ***G****AF****A****ssociated with Pil****Z***, considering its distinct structure and its strict occurrence as an insertion within PilZ domains (see more details below). Careful examination of secondary structure predictions revealed that the GAF-like domain is actually inserted into the PilZ domain after the first strand and the conserved RxxxR signature (Figure 1a,b). Structural models of LIC_11920 (YcgR_LIC_11920_) generated by AlphaFold (AlphaFold DB entry AF-Q72R31-F1) strongly suggest that the GAF-like domain within DUF1577 maintains its conformation without disrupting its own structural integrity or that of the PilZ domain (Figure 1b). The conserved residues among the YcgR-like paralogues of *L. interrogans* were mapped onto the three-dimensional structure model of YcgR_LIC_11920_, with most of them being in the PilZ domain (Figure 1b,c). Notably, the most highly conserved residues are the ones in the PilZ domain known to be involved in c-di-GMP binding (Figure 1a, motifs RxxxR and DxSxxG, marked by vertical lines).

### YcgR_LIC_11920_ Is a Monomer Due to the Lack of an α-Helix in the GAZ Domain Important for GAF Dimerization

**Figure 2:**
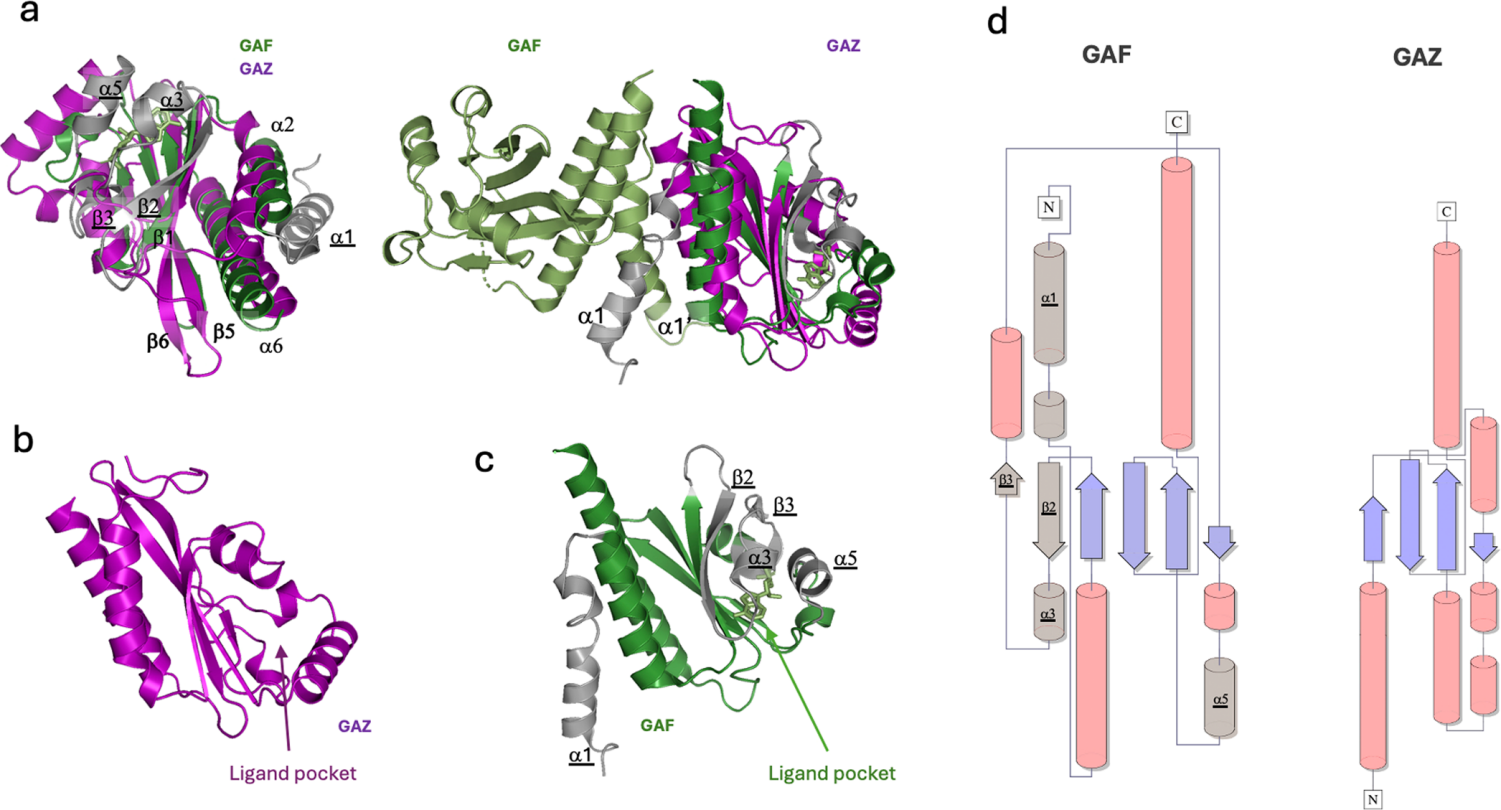
Structural analysis of the GAZ domain of YcgR_LIC_11920_. (a) Structure superposition of the heterodimer Lcd1 GAF domain (PDB 5W10,^4^ colored in light and dark green). The missing secondary structures in the GAZ domain are highlighted in gray in one of the Lcd1 molecules. The structural model of the GAZ domain of YcgR_LIC_11920_ is colored in purple in panels a and b. In panel b, the putative ligand pocket site of the YcgR_LIC_11920_ GAZ domain is indicated. Due to the absence of several secondary structures, this site is exposed to the solvent, in contrast to the more shielded ligand pocket observed in the Lcd1 GAF domain structure (panel c). (d) The topologies of the GAF and GAZ domains are shown. The missing secondary structures in the GAZ domain are highlighted in gray on the GAF domain topology.

Dimerization of GAF domains is often associated with signal transduction after binding of ligands and structural rearrangements to regulate the function of the other domains.^4,35−37^ Structural comparison of the GAZ domain of YcgR_LIC_11920_ to the canonical GAF domain of Lcd1 (PDB 5W10(4)) revealed several structural changes, the most important among them being the absence of YcgR_LIC_11920_ in the α-helix corresponding to α1 in Lcd1. This helix is required for Lcd1’s dimerization (Figure 2a), and previous studies have shown that the deletion of the first α-helix in other GAF domains significantly influences the oligomeric state, leading to the formation of both monomers and dimers in solution.^38^ These observations suggest that the insertion of the GAZ domain in YcgR_LIC_11920_ will not contribute to dimerization, and this protein might be expected to be monomeric, like other known YcgR homologues. Other secondary structure elements of Lcd1’s GAF that are absent in the GAZ domain are the α3 and α5 helices and the β2 and β3 strands. These elements are essential for maintaining the ligand-binding pocket closed in Lcd1 (Figure 2c), but in the GAZ domain of YcgR_LIC_11920_ this binding pocket is found to be exposed to the solvent (Figure 2b). Therefore, loss of secondary structure elements around the binding pocket gives the GAZ domain’s pocket a wider, more open conformation than most GAF homologues, but the implications of this feature are not yet elucidated (Figure 2a).

**Figure 3:**
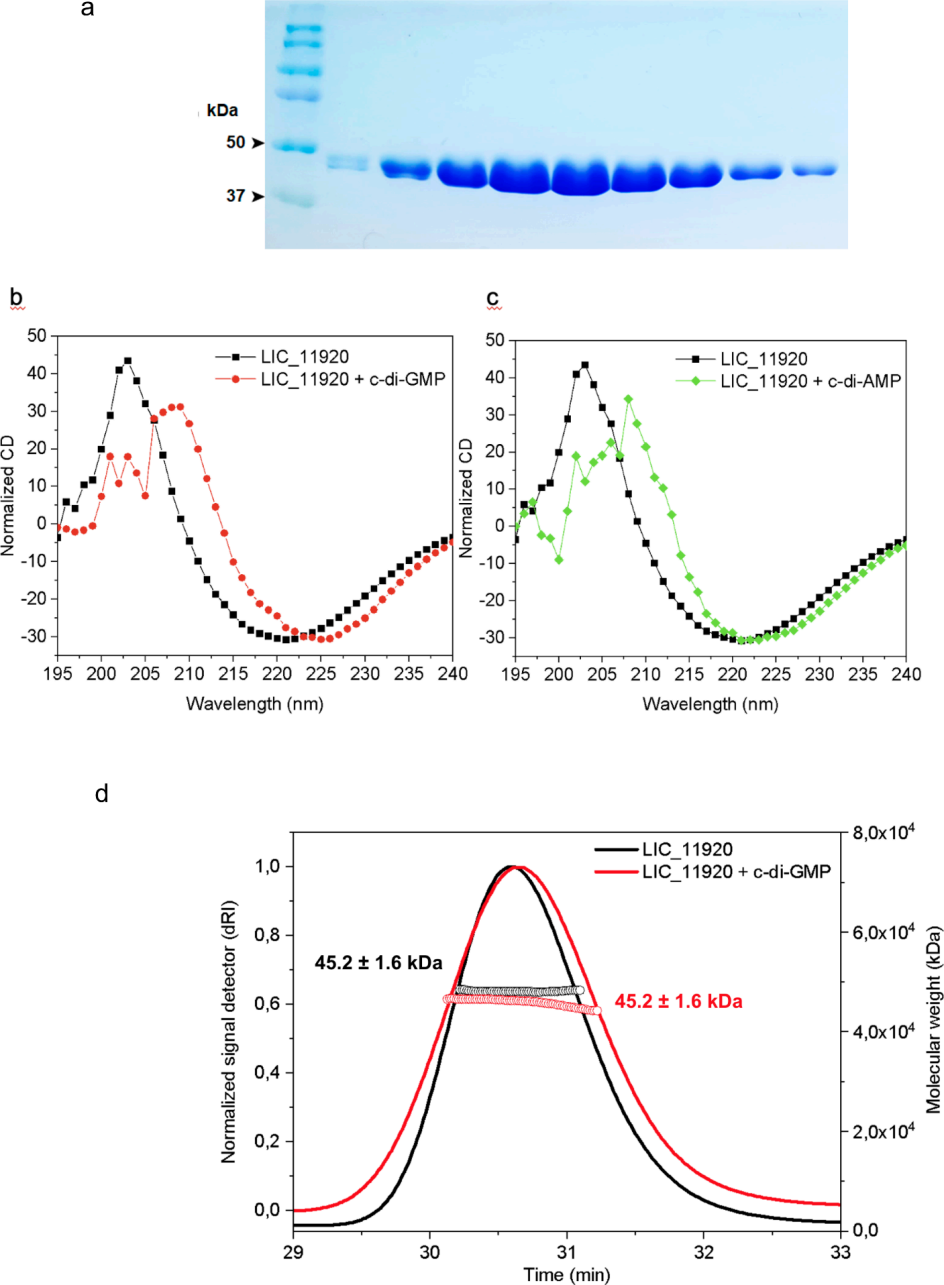
YcgR_LIC_11920_ is monomeric in the presence and in the absence of c-di-GMP by SEC-MALS assays. (a) 15% SDS-PAGE analysis of purified YcgR_LIC_11920_ following elution from a size-exclusion chromatography column. The theoretical molecular weight of YcgR_LIC_11920_ is 46 kDa. The first column in the SDS-PAGE gel corresponds to the Precision Plus Protein Standards kaleidoscope molecular weight marker (BIO-RAD). Circular dichroism profile of YcgR_LIC_11920_ in the presence and absence of cyclic dinucleotides: (b) YcgR_LIC_11920_ in the absence (black line) and presence of c-di-GMP (red line) and (c) YcgR_LIC_11920_ in the absence (red line) and presence of c-di-AMP (green line). In both cases, the protein is well folded. (d) Aliquots of ∼43 μM purified YcgR_LIC_11920_ in the absence (black line) and presence of 400 μM c-di-GMP (red line) were applied to a Superdex 200 increase 10/300 column. The lines represent the normalized refractive index (dRI), while the circles represent the molar weight distribution (kDa). YcgR_LIC_11920_ eluted as a monomer, showing molecular weights of 46 ± 2 and 45 ± 1.6 kDa in the absence (black) and presence of c-di-GMP (red), respectively. The replicates are listed in Figure S2.

To experimentally determine the oligomeric state of YcgR_LIC_11920,_ we cloned, expressed, and purified the full-length protein (Figure S1 and Figure 3a). The purified protein was well folded by circular dichroism (CD) analysis (Figure 3b,c). Size exclusion chromatography coupled with multiangle light scattering (SEC-MALS) assays were performed (Figure 3d), and our results showed that YcgR_LIC_11920_, in both the absence and presence of c-di-GMP, exhibited a monomeric profile with a slight conformational change observed in the triplicates (Figure S2). From the SEC-MALS assays, we determined an experimental molecular weight of 46 ± 2 kDa in the presence of c-di-GMP and 45.2 ± 1.6 in its absence (the theoretical molecular weight is 46.6 kDa, considering the 6x histidine tag at the N-terminal of the protein). Our findings support that GAZ is the first GAF-like domain not involved in dimerization.

### Conserved Residues in YcgR_LIC_11920_ Paralogs Correspond to c-di-GMP Binding Residues, While Conservation among Orthologs Suggests Novel Interaction Surfaces

**Figure 4:**
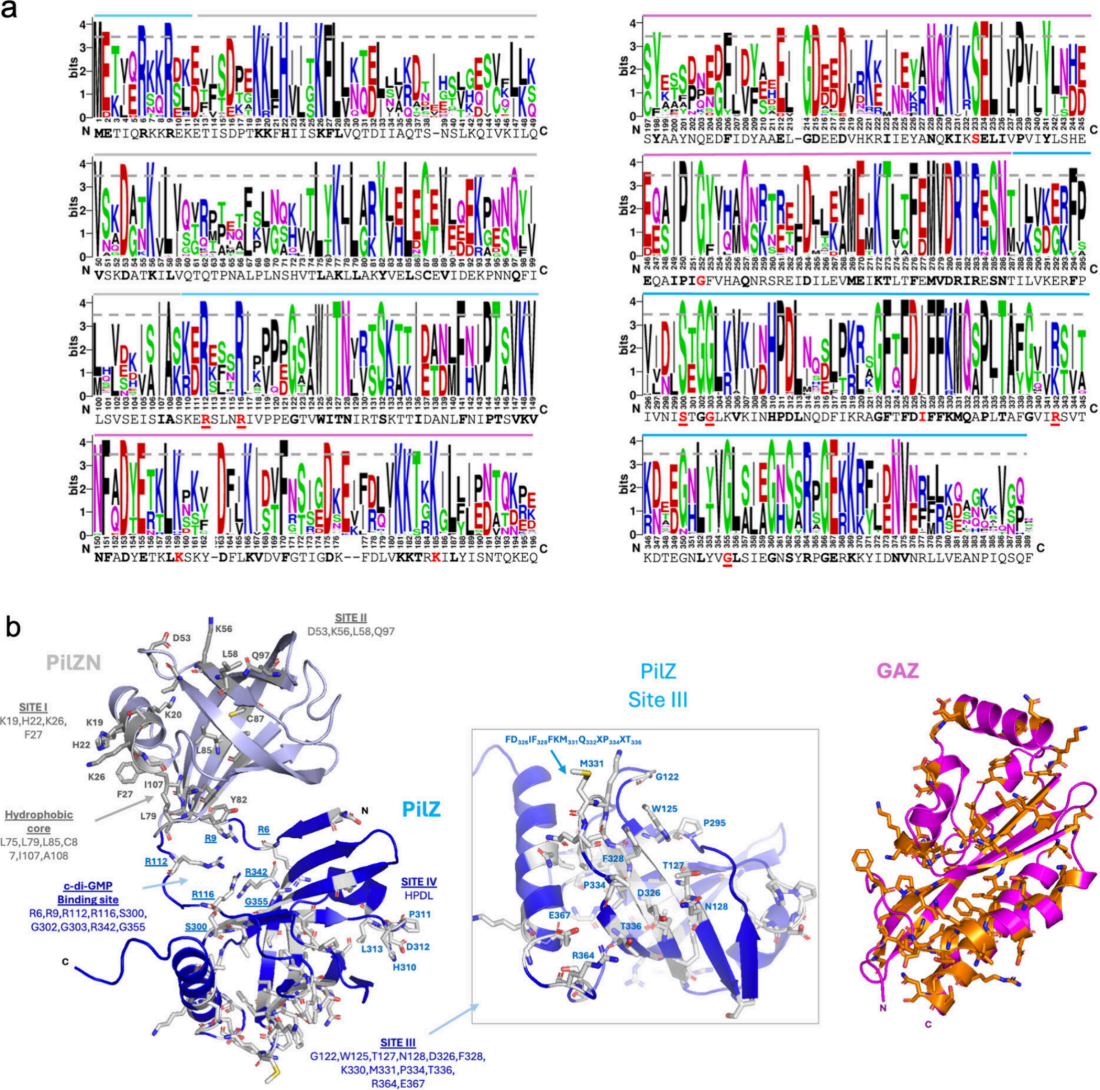
Amino acid residue conservation profile among YcgR_LIC_11920_ orthologs. (a) WebLogo representation of the amino acid residue conservation profile in the YcgR_LIC_11920_ orthologs. The dotted gray line indicates that residues above this line were considered conserved. The upper gray line represents residues that compose the PilZN domain; in blue, those that compose the PilZ domain; and in purple, those that compose the GAZ domain. The amino acid sequence below the Weblog belongs to the LIC_11920 protein, and those residues colored in red and highlighted are involved in c-di-GMP binding. The WebLogo was performed using a multiple sequence alignment performed by the COBALT server (computes a multiple protein sequence alignment using conserved domain and local sequence similarity information) with sequences searched using NCBI’s locus_tag LIC_11920 with percent identity between 40 and 100% and query coverage between 80 and 100%, excluding Models (XM/XP), Nonredundant RefSeq proteins (WP), and uncultured/environmental sample, and on the representative of each *Leptospira* specie. (b) The conserved residues are mapped in the AlphaFold2 model of YcgR_LIC_11920_, showing the PilZN domain (colored in gray), GAZ (colored in purple), and PilZ (colored in blue). Conserved residues are shown as sticks. We observed two conserved sites in the PilZN domain (Site I and Site II) and one hydrophobic core. In the PilZ domain, we observed a c-di-GMP binding site with more residues probably involved in the ligand binding and residues conserved in site III and site IV. In the GAZ domain, the conserved residues (colored in orange in the structure) are distributed in the structure, showing that this domain did not diverge in sequence enough to detect important residues for the functionality of the domain.

The multiple sequence alignment of all YcgR paralogues found in *L. interrogans* L1–130 (Figure 1c) reveals that all residues involved in c-di-GMP binding are still conserved in these proteins, despite insertion of the GAF domains between the conserved arginine residues and the DxSxxG motif.^39^ The multiple sequence alignment of a sample of YcgR_LIC_11920_ homologues enriched in orthologs is represented by a WebLogo (Figure 4a), and the conserved residues are mapped in the AlphaFold2 structure model of YcgR_LIC_11920_ (Figure 4b). Within the PilZN domain, we identified two conserved sites, Site I and Site II, along with a hydrophobic core. In the PilZ domain, we identified two sites, Site III and Site IV, located on one face of the β-sheet barrel. In the c-di-GMP binding site, we identified more positively charged residues that may contribute to ligand binding. Interestingly, two of them, R6 and R9, are located at the amino-terminal region of the protein in a β-strand that probably contributes to keep the PilZN more tightly connected with PilZ (Figure 4b). In the GAZ domain, the conserved residues are distributed throughout the structure, indicating that this domain did not accumulate enough variation to allow the identification of the residues that are most relevant to its function. Since the exposed residues in the sites I to IV (Figure 4b) are located in surface exposed areas of the PilZN and PilZ domains, it is possible that these sites are involved in protein–protein interactions. In this regard, several PilZN and PilZ domains have already been shown to participate in protein–protein interactions, such as the YcgR-c-di-GMP of *E. coli* which binds to the stator protein MotA through its PilZ domain and to FliG through its PilZN domain, thereby regulating bacterial swimming.^24^ Another example is the noncanonical PilZ that binds PilB and FimX in *X. citri* to regulate twitching motility.^17,40^ The differences in the sequence conservation profiles of YcgR_LIC_11920_ orthologs (Figure 4a) and YcgR_LIC_11920_ paralogs (Figure 1c) suggest that the YcgR_LIC_11920_ paralog family may have been selected to keep the c-di-GMP binding activity while acquiring new protein interactions.

### YcgR_LIC_11920_ Binds c-di-GMP Despite the Insertion of a GAZ Domain within the PilZ Domain

To determine whether YcgR_LIC_11920_ can in fact bind c-di-GMP we performed a series of experiments, including circular dichroism (CD) and isothermal titration calorimetry (ITC). The CD spectrum profile of YcgR_LIC_11920_ in the absence and presence of c-di-GMP showed a small shift at 220 nm (Figure 3a), indicating that the binding of c-di-GMP by YcgR_LIC_11920_ might cause a secondary structural change in the protein. In contrast, the presence of c-di-AMP did not induce a significant change in the CD spectrum profile (Figure 3b).

To evaluate the thermal stability of YcgR_LIC_11920_ in the presence and absence of c-di-GMP, c-di-AMP, GTP, and different cations such as Ca^2+^ and Mg^2+^, we monitored the thermal denaturation of the protein by CD at 230 nm. The results showed no significant gain in structural thermal stability with c-di-GMP, c-di-AMP, GTP, and Mg^2+^ (Figure S3). In contrast, a slight increase in the melting temperature (Tm) of approximately 1 °C was detected in the presence of calcium chloride (CaCl_2_), indicating a potential calcium-binding capability. Thus, our thermal stability data suggest that if YcgR_LIC_11920_ is a c-di-GMP receptor interaction with c-di-GMP does not notably enhance the protein’s thermal stability.

**Figure 5:**
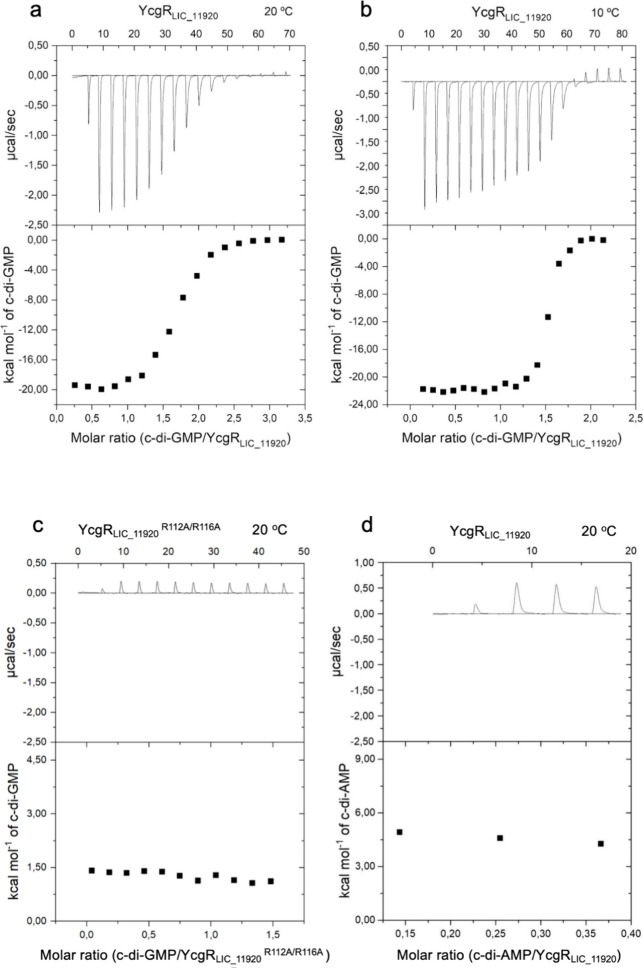
YcgR_LIC_11920_ selectively binds to c-di-GMP by ITC assay. Exothermic profile of interaction between YcgR_LIC_11920_ and c-di-GMP at 20 °C (a) and 10 °C (b). Two biological replicates were performed (Figure S5). (c) YcgR_LIC_11920_ mutant for residues R112A and R116A showed no interaction with c-di_GMP at 20 °C. (d) YcgR_LIC_11920_ did not interact with c-di-AMP at 10 °C as shown by an endothermic profile, consistent with the dilution of c-di-AMP.

We then used ITC experiments in order to detect the interaction of c-di-GMP to YcgR_LIC_11920_. Titrating c-di-GMP into the YcgR_LIC_11920_ solution revealed a pronounced release of heat upon binding (Figure 5) and a stoichiometric c-di-GMP:YcgR_LIC_11920_ ratio of 2:1 (Table 1). The same stoichiometry is observed for the YcgR homologue FilZ protein of *Pseudoalteromonas* sp.^41^ and YcgR of *E. coli*.^42^ Fitting binding isotherms of a one binding site model results in estimates for the dissociation constant *K*_D_ of 60 nM at 10 °C and 300 nM at 20 °C, which correspond to free energy changes (Δ*G°*) of −9.2 ± 0.2 kcal mol^–1^ and −8.8 ± 0.3 kcal mol^–1^ (Δ*G°* = *RT* ln *K*_D_) (Table 1). The corresponding entropy change values are −12.6 ± 0.1 kcal mol^–1^ K^–1^ and −10.8 ± 0.2 kcal mol^–1^ K^–1^ at 10 and 20 °C, respectively (Table 1). Notably, a comparable value at 25 °C was reported in the interaction between c-di-GMP and YcgR from *E. coli*.^42^ The binding affinities of c-di-GMP for protein receptors vary over several orders of magnitude with values ranging from approximately 5 nM to 50 μM.^43,44^ As expected, the interactions between the PilZ domain and c-di-GMP are mediated by mainly ionic contacts and polar interactions through hydrogen bonding involving the arginines in the RxxxR and DxSxxG motifs. Interestingly, preliminary results for the variation of Δ*H* with temperature show a positive heat capacity change (Δ*C*_*p*_) of +220 ± 10 cal K^–1^ mol^–1^ for the interaction between YcgR_LIC_11920_ and c-di-GMP. This positive change in Δ*C*_*p*_ is typically associated with the burial of polar surfaces as a result of the interaction.^45^ Interaction assays with YcgR_LIC_11920_ mutants, where the arginines in the c-di-GMP interaction motif (R^112^xxxR^116^) were substituted for alanines, showed a loss of interaction with the ligand (Figure 5c). This suggests that YcgR_LIC_11920_ binds to c-di-GMP in a manner similar to that of other PilZ domains. The interaction of YcgR_LIC_11920_ with c-di-AMP was not observed in ITC assays (Figure 5d) indicative of the specificity for c-di-GMP.

**Table 1:** YcgR_LIC_11920_ Binding Parameters to c-di-GMP Determined by ITC Assays

Protein	Temperature	Δ*H*, kcal mol^–1^	*T*Δ*S*, kcal mol^–1^	*K*_D_, nM	Δ*G*, kcal mol^–1^	c-di-GMP:YcgR_LIC_11920_ ratio
YcgR_LIC_11920_	10 °C	–21.8 ± 0.1	–12.6 ± 0.1	60	–9.2 ± 0.2	2:1
YcgR_LIC_11920_	20 °C	–19.6 ± 0.1	–10.8 ± 0.2	300	–8.8 ± 0.3	2:1

The remarkably exothermic nature of the interaction observed between c-di-GMP and YcgR_LIC_11920_ suggests that conformational changes in binding impact the structure beyond the binding pocket. Previous investigations employing FRET analyses^46^ and NMR spectroscopy^47^ have indeed shown conformational changes in various PilZ domains upon c-di-GMP binding. The significant entropic penalty suggests a loss of conformational flexibility, likely due to conformational restrictions imposed by c-di-GMP binding. In summary, the thermodynamic parameters of YcgR_LIC_11920_ c-di-GMP binding reveal an enthalpically driven binding process with entropic penalties as observed in other proteins containing PilZ domains.^17,41,44,48−54^

A noteworthy feature of c-di-GMP as a ligand is its ability to assume various oligomeric equilibrium states, leading to significant variations in binding stoichiometry across receptors in the ITC experiments.^43,55^ The stoichiometry of c-di-GMP binding to PlzA exhibits a 1:1 ratio (ligand to receptor) with an affinity of 6.3 ± 1.1 μM.^46^ In contrast, the wild-type AxCeSA-PilZ binds two molecules of c-di-GMP with nearly identical affinity of 6.0 μM.^50^ In our ITC assays, we kept c-di-GMP at 60 °C for 1 h before the experiment for maximum monodispersity of the dinucleotide.

c-di-GMP binds to its receptors in diverse oligomeric configurations, such as monomeric, dimeric, trimeric, and tetrameric.^21^ In this study, the binding stoichiometry was experimentally determined to be 2:1 (c-di-GMP:YcgR_LIC_11920_). Our ITC data fit the predictions of a one binding site model for the 2:1 stoichiometry.^43^ The c-di-GMP dimer is slightly favored over its dissociated form (c-di-GMP monomer), as the energy difference between these two conformations is only a few kilocalories per mole.^56^ Since c-di-GMP is in equilibrium with different oligomeric states, the protein probably binds in a single step as the more favorable c-di-GMP conformation to be bound into its binding site. The binding stoichiometry of 2:1 may mean either a dimer of c-di-GMP to a YcgR_LIC_11920_ monomer or 4 c-di-GMP to a dimer of YcgR_LIC_11920_, and SEC-MALS assays showed that YcgR_LIC_11920_ is a monomer in the absence and presence of c-di-GMP (Figure 3). Therefore, our results suggest that one molecule of YcgR_LIC_11920_ binds to two c-di-GMP molecules. We do not have enough data to distinguish if YcgR_LIC_11920_ binds one molecule of c-di-GMP and another to a c-di-GMP dimer or directly a dimer of c-di-GMP.

### Molecular Docking and Molecular Dynamics Simulations of the YcgR_LIC_11920_ and c-di-GMP Complex

**Figure 6:**
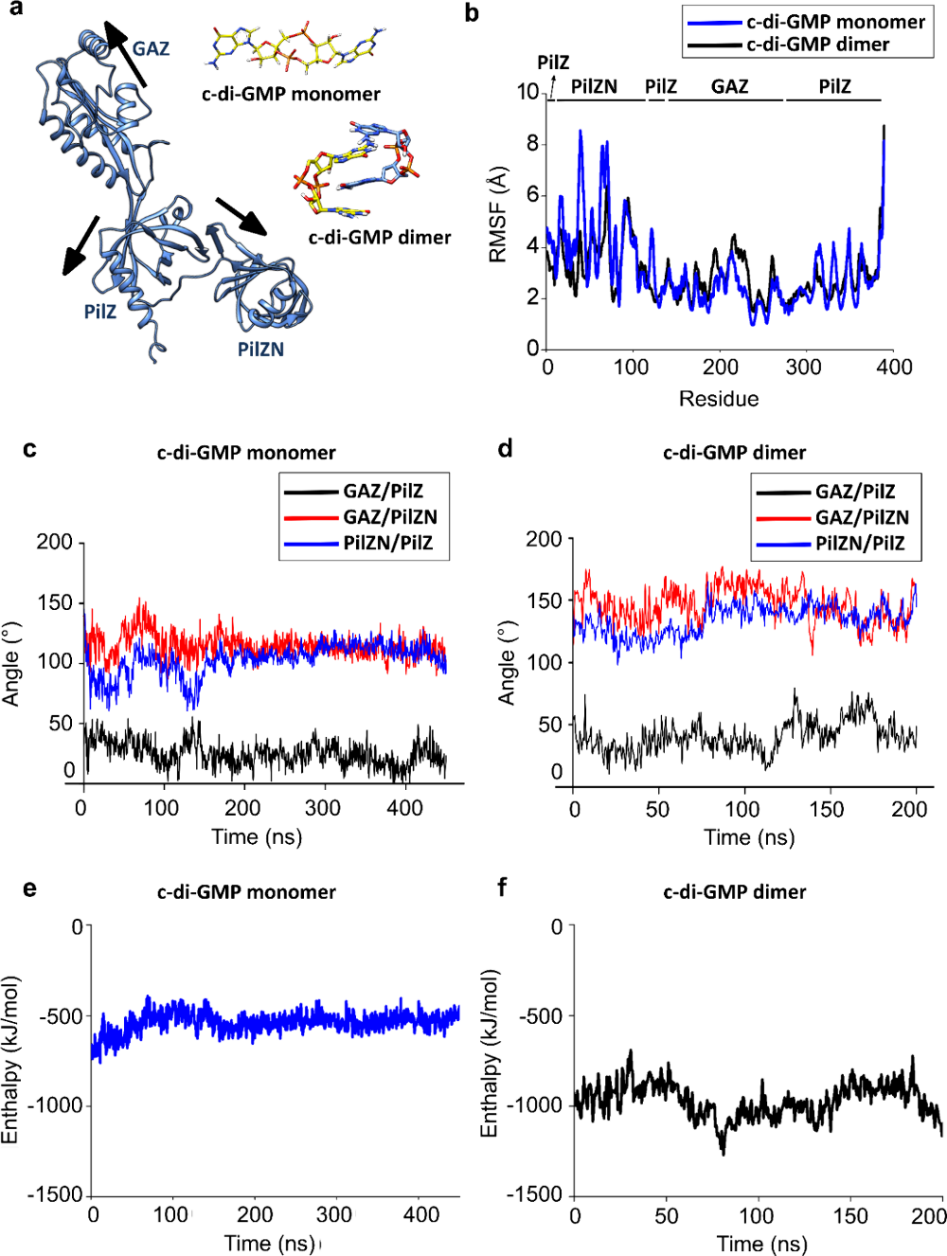
Molecular docking and molecular dynamics of YcgR_LIC_11920_ in a complex with a monomer and dimer of c-di-GMP. (a) Molecular docking simulation of a monomer and dimer of c-di-GMP. The top pose presented a docking score of −10.6 and −9.7 kcal mol^–1^ for a monomer and dimer of c-di-GMP, respectively. The arrows are vectors relative to the center of mass of each domain. In this regard, we analyzed the amino acid residues comprising these domains as follows: PILZ has residues 1–12, 110–149, and 288–389, PILZN has residues 13–109, and GAZ has residues 150–287. These vectors (for each frame) were calculated by considering a sum of difference between the center of mass of each domain (reference) and spatial coordinates of each atom of this domain (without normalization). (b) Backbone root-mean-square fluctuation (RMSF) of each residue of YcgR_LIC_11920_ with the monomer (blue) and dimer (black) of c-di-GMP. Note that high structural flexibility is localized in PilZN and PilZ, when compared with GAZ. However, the GAZ domain presents more flexibility when the c-di-GMP dimer interacts with YcgR_LIC_11920_ than with the c-di-GMP monomer. Angles between representative resultant vectors of each domain (PilZN, GAZ, and PilZ) along with time for YcgR to interact with the (c) c-di-GMP monomer and (d) c-di-GMP dimer. Enthalpy was calculated from MM-PBSA from the molecular dynamics trajectory when YcgR_LIC_11920_ interacts with the (e) c-di-GMP monomer and (f) c-di-GMP dimer.

To investigate how the insertion of the GAZ domain influences the binding mode of YcgR^GAZ^ to c-di-GMP and its interactions with the PilZN and PilZ domains, we ran molecular dynamics simulations of the apo and holo states based on the YcgR_LIC_11920_ structure predicted by AlphaFold. Our experimental data indicated that YcgR_LIC_11920_ is a monomer capable of binding a dimer of c-di-GMP (Figures 3 and 5). We therefore chose to construct two holo state systems, one docking the c-di-GMP monomer (Figure S5) and another with the c-di-GMP dimer (based on the structure with PDB 5Y6F(24)) docked into the binding site. Docking of the monomer and the dimer of c-di-GMP at the binding site of YcgR_LIC_11920_ resulted in free energies of ∼−9.6 and ∼9.7 kcal mol^–1^, respectively. These values are similar to those obtained in ITC assays (Table 1). These two holo systems were then used as initial states for the molecular dynamics simulations. We simulated both complexes until they converged, resulting in simulation times of 450 and 200 ns, respectively (Figures 6b–d and S6 and movies S1 and S2). The RMSD for the monomer interaction changed from ∼0.6 to ∼0.7 nm in ∼150 ns, while for the dimer interaction it altered from ∼0.5 to ∼0.7 nm in ∼80 ns. Both simulations converged to an RMSD of approximately 0.7 nm (Figure S6a and c). For YcgR_LIC_11920_, whether interacting with the monomer or dimer of c-di-GMP, a structural change occurred due to the rearrangement of the binding site to accommodate the c-di-GMP. These structural changes in the presence of the ligand are consistent with the CD data (Figure 3a).

The initial analysis aimed to verify the radius of gyration over time to examine the molecular weight distribution around the center of mass of YcgR_LIC_11920_ interacting with the monomer and dimer of c-di-GMP (Figure S6b and d). The radius of gyration is often used to characterize the geometry and shape of molecules, which is particularly useful in characterizing the degree of protein compactness. For YcgR_LIC_11920_ interacting with the c-di-GMP monomer or dimer, the MD simulation results in a radius of gyration that is stable for Rg, Rgx, Rgy, and Rgz, with similar values in both cases (Figure S6b and S6d).

Our MM-PBSA results showed that the YcgR_LIC_11920_/c-di-GMP monomer presented an average enthalpy value of −539 ± 56 kJ mol^–1^, while the YcgR_LIC_11920_/c-di-GMP dimer showed a value of −969 ± 94 kJ mol^–1^ (Figure 6e and f). This shift of the enthalpy occurs because binding to the c-di-GMP monomer induces conformational changes in YcgR_LIC_11920_ that result in a distance of approximately 15 Å between R112 and R116 (Figure S7a and b). In the case of the YcgR_LIC_11920_/c-di-GMP dimer these arginines are separated by ∼5 Å (Figure S7b). In both complexes, the R116 residue plays a key role in the interaction with the phosphate group of c-di-GMP, as indicated by the distances from this residue to the phosphate group (Figure S7c and d). The distance between K110 to the phosphate group of the c-di-GMP monomer is ∼5 Å (Figure S7e).

In our simulation, the interaction of YcgR_LIC_11920_ and the c-di-GMP dimer is mediated predominantly by residues D32, D42, D112, and R116, with hydrogen bonding occupancies of 77, 59, 68 and 85%, respectively. One molecule of c-di-GMP interacts with PilZN residues (D32 and K42). Interestingly, position 32 is a conserved acid residue (D/E) in YcgR_LIC_11920_ orthologs (Figure 4a). The other c-di-GMP molecule exposes phosphate groups that interact with conserved arginines of the PilZ domain (R112 and R116) (Figure S7d and f). These arginine interactions are consistent with our experimental data, which shows that mutations in these two arginines abolish the protein’s ability to bind c-di-GMP (Figure 5c).

In order to investigate the relative position of each domain, we calculated the average vector relative to the center of mass for each domain and monitored the angles between these vectors (Figure 6c and d). The angles between GAZ/PilZN and GAZ/PilZ are both larger for the YcgR_LIC_11920_/c-di-GMP dimer than the respective angles measured for the YcgR_LIC_11920_/c-di-GMP monomer. The higher RMSF values of the amino acid residues in the PilZN and PilZ domains of the YcgR_LIC_11920_/c-di-GMP monomer compared to the YcgR_LIC_11920_/c-di-GMP dimer (Figure 6b) show that the latter complex possesses a more stable tertiary structure along the MD simulation.

**Figure 7:**
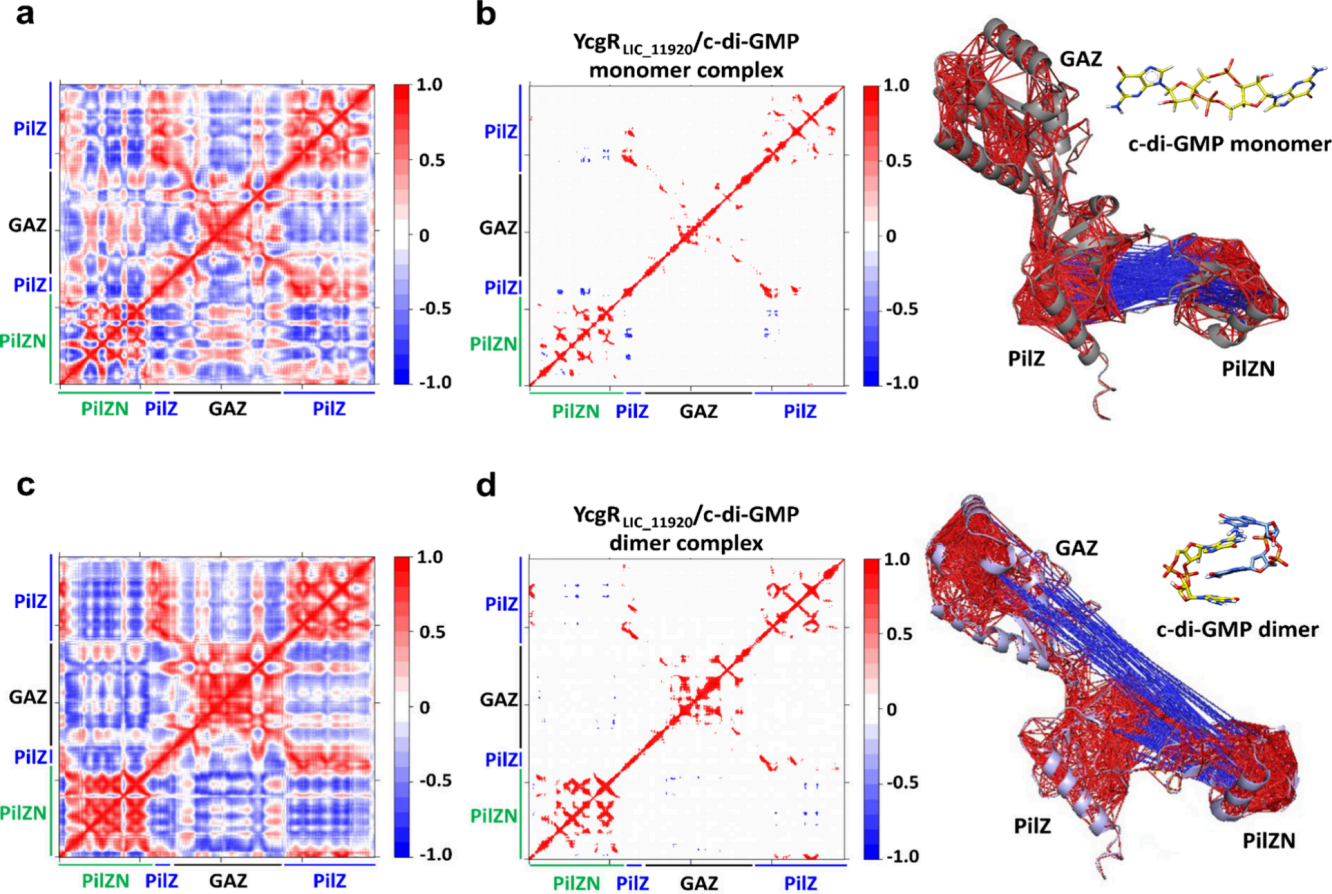
Dynamic cross-correlation matrix (DCCM) projected onto the structure of the LIC_11920 interacting with the monomer and dimer of c-di-GMP. Correlation and anticorrelations are represented by red and blue, respectively. (a) Full correlation matrix without filtering. (b) Filtered correlation matrix, where correlations between −0.8 and 0.8 were removed. Projection of the correlations (*r* > 0.8, in red) and anticorrelations (r < −0.8, in blue) onto the 3D structure of the YcgR_LIC_11920_ while interacting with the c-di-GMP monomer. The anticorrelations were observed only between PilZN and PilZ colored in blue. (c) Full correlation matrix without filtering. (d) Filtered correlation matrix, where correlations between −0.8 and 0.8 were removed. Projection of the correlations (*r* > 0.8, in red) and anticorrelations (*r* < −0.8, in blue) onto the 3D structure of the YcgR_LIC_11920_ while interacting with the c-di-GMP dimer. The anticorrelations, indicated in blue, were observed between PilZN and PilZ, as well as between GAZ and PilZ.

We also calculated the displacement cross-correlation matrix (DCCM) by using the simulated trajectory and focused on strong correlations and anticorrelations (Figure 7). In all simulations, we observed strong correlations between the PilZN and PilZ domains. The MD simulations for the YcgR_LIC_11920_ with the c-di-GMP monomer also show that, in this system, the GAZ domain maintains only internal correlations within itself (Figure 7b). This result contrasts with the presence of strong anticorrelations between PilZN and GAZ for the dimer simulations (Figure 7d). This suggests that movements in the PilZN domain are sensed by the GAZ domain.

### GAZ Insertions in YcgR Homologues Are Widespread among Spirochaetes

Using Pfam’s DUF1577 and custom Hidden Markov Models, we searched for members of the new YcgR family in the NCBI’s nonredundant protein database (NR). To facilitate understanding, from here on in the text, YcgR homologues with the domain architecture observed for YcgR_LIC_11920_ will be referred to as YcgR^GAZ^. This search recovered proteins from genomes of several spirochetes beyond *Leptospira*, including the Leptospirales *Turneriella* and *Leptonema*, the hot-spring anaerobic *Thermospira aquatica*, a member of the Brevinematales order, and several members of the Spirochaetales order, such as *Marispirochaeta*, *Salinispira*, *Brucepastera*, and *Treponema primitia*. Multiple paralogs of YcgR^GAZ^ were found in genomes of all members of the Leptospirales order, with the number of full-length homologues varying between three and eight genes per genome (Supporting Information File S1). In contrast, *Thermospira aquatica* has only two paralogs of YcgR^GAZ^ and no other YcgR-like protein; however, in Spirochaetales one or two YcgR^GAZ^ homologues may occur, but other YcgR family members usually occur.

Additional searches for GAZ domains, using iterative methods, could not identify any homologues outside of the phylum Spirochaetota. To search for additional YcgR homologues that could be related to the YcgR proteins with GAZ insertions, we characterized the taxonomic distribution of YcgR homologues and PilZ-like domains by searching for these domains in the NR database. In agreement with previous surveys,^18,57^ our results revealed the presence of a diverse repertoire of PilZ-like genes in several bacterial genomes (Supporting Information File S1). While some bacteria were found to have only one PilZ-like protein, such as *Borrelielia* and *Bacillus subtilis*, the number of PilZ-like genes could be as high as 33, as seen in *Nitrospira moscoviensis* (Supporting Information File S1). Several of the proteins identified in these searches belong to well-known PilZ families, such as the cellulose synthase fusions that were the first c-di-GMP binding proteins to be identified. Standalone PilZ domains were the most common protein domain architectures in our data set, but the second most common architectures were composed by two PilZ-like domains, including the classical YcgR architecture, made up of a divergent N-terminal PilZ-like domain (PilZN) and a C-terminal PilZ domain with the conserved c-di-GMP binding motifs RxxxR and [D/N]hSxxG.^18^ Other examples of proteins with two consecutive PilZ-like domains included those with two copies of the PilZ domain, recognizable as hits of the models for PilZ domains from Pfam (accession numbers PF07238 and PF16823) and by the presence of the binding motifs in both copies. More divergent examples of members of the PilZ-like family, having up to three PilZ-like domains in tandem, displayed different combinations of domains with and without binding motifs (Supporting Information File S1).

The high degree of divergence observed for many YcgR homologues challenges identification of PilZN domains by pHMMs found in public databases. Careful inspection of the predicted structures of proteins identified by PilZ models revealed the presence of many unidentified PilZ-like domains preceding homologues of the PilZ domain. Several of these domains are divergent versions of known PilZN families, such as PilZN3, while others are most likely new families. The high degree of sequence divergence among the N-terminal PilZ-like domains and the lack of unifying structural features could imply independent origins of these subfamilies, with N-terminal versions of PilZ evolving from different branches of the PilZ-like family. We therefore chose to designate all sequences carrying an N-terminal PilZ-like domain fused to a recognizable C-terminal PilZ domain as YcgR-like proteins, regardless of whether these sequences actually belong to a monophyletic group. Among these results, some YcgR-like proteins from Myxococcota, Deltaproteobacteria, and other lineages (clusters MBI4594913.1 and MCA9504126.1 in Supporting Information File S2) were found to contain extended regions in the loop between the first and second strands of the C-terminal PilZ domain. These regions are predicted to contain one to three α-helices (AlphaFoldDB: AF-A0A7 V6C6Z0-F1), and although such insertions induce more pronounced distortions in the second strand of the PilZ domain than the GAZ domain, they do not seem to prevent proper folding of the PilZ β-barrel. The binding motifs are preserved in several members of this group. Notably, very similar insertions were previously shown to be a feature responsible for oligomerization of the tetrameric tPilZ subfamily (PDB IDs:3RQA and 3PH1), but the tPilZ family is a family of standalone PilZ domains and therefore not a YcgR-like protein.^18^

Examination of the multiple copies of YcgR-like proteins that include YcgR^GAZ^ and other insertions, from Spirochaetales, also revealed the presence of an N-terminal extension previously described by Galperin and Chou as a pentahelical subdomain,^18^ which we named NpzN, for “**N**-terminal to **P**il**ZN**”. YcgR homologues containing NpzN domains, which we here name as YcgR^NpzN^, have a conserved domain architecture comprising a long transmembrane helix, followed by the NpzN domain and the classical PilZN and PilZ domains characteristic of the YcgR family (Figure S8). The NpzN domain is absent in all YcgR^GAZ^ proteins, and although some members of the GAZ subfamily are present in Spirochaetales alongside NpzN homologues, such as *Treponema*, *Salinispira*, and *Spirochaeta*, NpzN domains were not easily identified in members of the Leptospirales. Although initially described as a subdomain, NpzN exhibits structural similarities with the matrix domain (MA) of the Rous sarcoma virus Gag protein, as both regions pack four of their α-helices as pairs that pile up perpendicularly on top of each other (Figure S8). This resemblance suggests a potential role for NpzN in mediating interactions with components of the bacterial cytoplasmic membrane. We also observed several examples of α-helical N-terminal extensions in YcgR-like proteins that did not produce significant alignments against our HMM model for the NpzN domain. These other proteins, although unrelated to NpzN, also have at least one hydrophobic α-helix, predicted to be a transmembrane region, followed by two to five parallel and/or antiparallel helices arranged as a bundle. Given their similar contexts, both the NpzN domain and these other extensions may be involved in protein–protein and/or protein–membrane interactions.

Given the extreme diversity of our sample of YcgR-like proteins, which includes YcgR^GAZ^ and YcgR^NpzN^, we sought to evaluate whether associations of YcgR-like proteins with other gene families could provide evidence for functional links. We collected 11,980 gene neighborhoods for a nonredundant subset (sequence identity less than or equal to 70%) of the YcgR-like proteins. These loci included up to seven upstream and seven downstream genes of the canonical YcgR, YcgR^GAZ^, and YcgR^NpzN^ homologues. We found that 3433 of these loci contained at least one gene involved in the flagellum assembly. Considering a binomial model for independent sampling and an estimate of an average of 60 flagellum genes per genome, these results indicate that flagellar genes occur more frequently than expected near loci of YcgR-like proteins (*p-value* ≤ 4.5 × 10^–59^). This association is congruent with the known role of YcgR as a flagellum regulator. It is even stronger for the known interaction partners of YcgR, MotA, and MotB, which were found in 202 of the sampled loci (*p*-value = 0), while 100 of these loci had both a flagellar motor protein and at least one other flagellum marker gene.

Using the census of YcgR homologues, we assigned all homologues to five subfamilies, canonical YcgR, YcgR^NpzN^, YcgR^GAZ^, PilZN, and PilZ. Canonical YcgR members were recognized, as described above, by the presence of an N-terminal PilZ-like divergent domain (PilZN) and a C-terminal PilZ domain. Recognition of the GAZ and NpzN domains by our custom models was used to assign proteins to the YcgR^GAZ^ and YcgR^NpzN^ subfamilies. The last subfamily is the PilZN subfamily corresponding to proteins with divergent PilZ-like domains and those without PilZ domains. All of the other proteins with PilZ domains were assigned to the PilZ subfamily. We then evaluated the Pearson correlations for the number of genes in each subfamily across all genomes in our data set. These results are shown in Tables S2–S3. When considering all genomes where PilZN proteins were detected, Pearson coefficients between the YcgR^GAZ^, YcgR^NpzN^, YcgR, and PilZ subfamilies are nonsignificant (*r* ≪ 0.85), but the YcgR^GAZ^ and YcgR subfamilies are negatively correlated if only genomes from the Spirochaetota phylum are considered (*r* = −0.76). At the same time, PilZ abundance is positively correlated with that of the YcgR^GAZ^ family. These results suggest that although YcgR^GAZ^ proteins and YcgR may be found in the same genomes the YcgR^GAZ^ subfamily expansion could result in replacement of YcgR proteins without GAZ insertions by the new subfamily. Whether this possibility actually corresponds to one or more of the YcgR^GAZ^ paralogues taking over the role that the canonical YcgR orthologs play remains to be determined.

**Figure 8:**
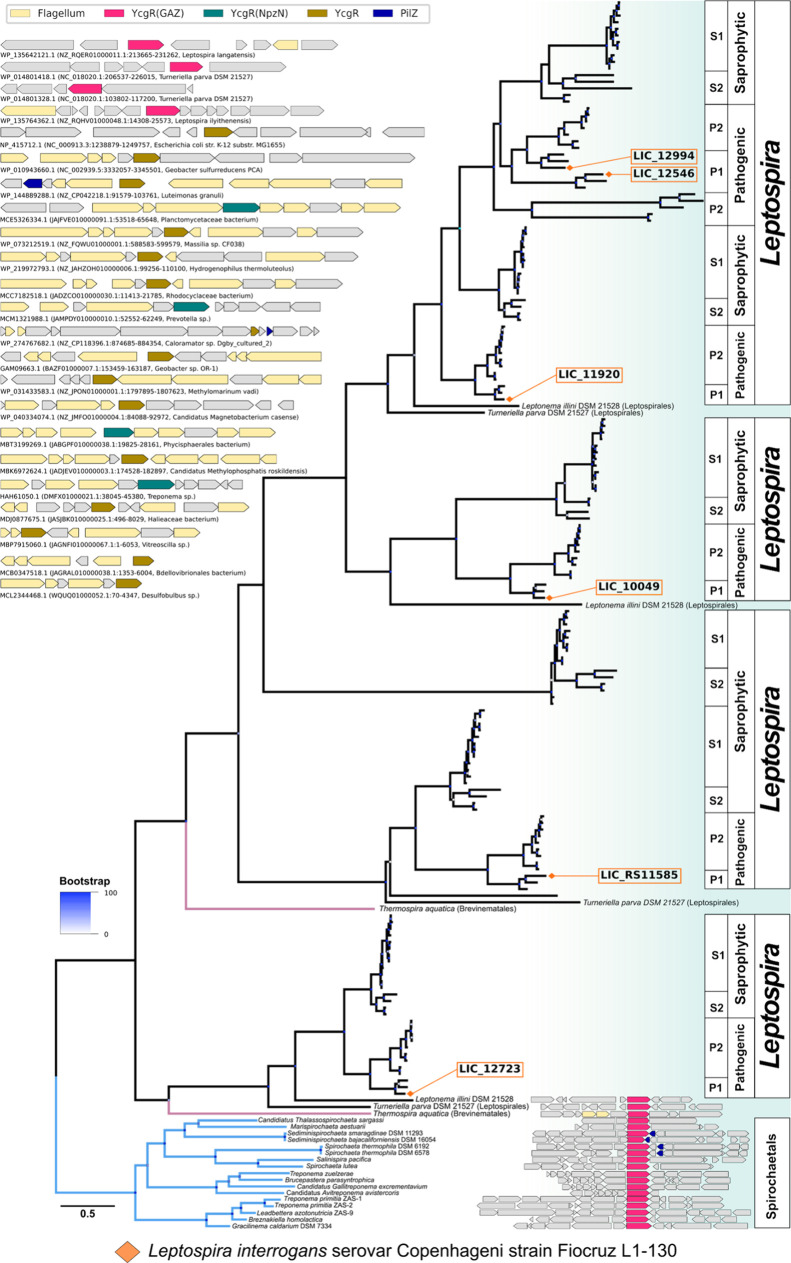
Phylogeny and genomic context of YcgR homologues. The upper left and lower right panels show gene neighborhoods for YcgR homologues (colored in pink) collected from several bacterial lineages. Flagellar genes (yellow) are rare near members of the YcgR^GAZ^ subfamily. At least six events of gene duplication that led to the expansion of the YcgR^GAZ^ subfamily can be inferred from tree topology. The phylogeny was inferred using maximum likelihood methods implemented in IQtree, version 2.3.3.

To understand the events that gave origin to the paralogues of the YcgR^GAZ^ subfamily, we collected representative members of this subfamily from complete genomes and built a phylogenetic tree using maximum likelihood methods (Figure 8). Although the high rates of evolution affecting this family affect the phylogenetic signal contained in the alignment, several internal nodes were recovered with high levels of support, and the tree topology mostly reproduces the phylogeny of the species in our sample, suggesting a pattern of vertical inheritance with no signs of lateral gene transfer. The phylogeny also indicates that expansion of the YcgR^GAZ^ subfamily started before or alongside the divergence of most lineages within the Spirochaetia class, with one such event predating the divergence of Brevinematales and Leptospirales. Two other duplication events date back to at least the most recent common ancestor of all Leptospirales, leading to the presence of three paralogues in *Turneriella* and *Leptonema*. Further duplication events, inferred to have occurred after the emergence of *Leptospira*, gave origin to additional paralogues found only in this species. The phylogeny shows that several of the YcgR^GAZ^ lineages exclusive to either saprophytic or pathogenic *Leptospira* would, under a most parsimonious scenario, have first appeared in the ancestor of these two lineages, followed by losses in the extant lineages that do not have them. Alternatively, accelerated rates of evolution of the most divergent YcgR^GAZ^ copies in lineages of *Leptospira* might have followed their emergence and caused the wrong placement of these lineages in the phylogeny.

## Discussion

In this study, we showed that *L. interrogans* has six divergent paralogues of the YcgR protein family, all of which are characterized by the insertion of a GAF-like domain into the PilZ domain, representing a novel domain architecture for members of the YcgR protein family. The GAF domain was originally identified by Aravind and Ponting in 1997,^58^ and its name is an acronym formed by the initials of the names of the three protein classes where versions of this domain were first identified: mammalian cGMP-binding PDEs, *Anabaena*adenylyl cyclases, and *Escherichia coli*FhlA.^58,59^ GAF domains are present in different types of proteins and are associated with light absorption and ethylene signal transduction in plants and with gene regulation via two-component systems in bacteria and archaea. Interestingly, several of these domains have been shown to bind cyclic nucleotides such as cAMP and cGMP.^60^ Sequence searches and analysis of computationally derived structures of the *L. interrogans* serovar Copenhageni’s protein YcgR_LIC_11920_ and its paralogs support the assignment of the GAF-like insertion to a novel GAF family, which we named GAZ. Importantly, GAF domains usually form dimers with the help of a conserved helix, but deletion of this helix and other conserved secondary structure elements in the GAZ domain prevents YcgR_LIC_11920_ dimerization.

Although PilZ domains are extremely diverse, the only known example of an insert into the split β-barrel of this fold’s core is found in the atypical tetrameric PilZ of XCC6012 (PDB:3RQA). Interestingly, the long α-helices inserted into XCC6012’s PilZ domain are located at the same position as the GAZ domain insert of YcgR^GAZ^, between the first and second strand of the barrel, but XCC6012 forms tetramers that do not bind c-di-GMP. We evaluated the binding properties of the YcgR_LIC_11920_ protein using different biochemical assays and found that this protein is a *bonna fide* c-di-GMP receptor. ITC experiments revealed a 2:1 c-di-GMP:YcgR^GAZ^ stoichiometry and a pronounced heat release upon binding. YcgR_LIC_11920_ binds c-di-GMP with dissociation constants ranging from 60 nM at 10 °C to 300 nM at 20 °C, indicating a strong binding affinity primarily driven by enthalpy changes. As expected, replacement of the key arginines (R112 and R116) in the conserved RxxxR motif of the PilZ domain by alanines abolished binding, and no evidence of interaction with c-di-AMP was observed for the YcgR_LIC_11920_ wild type. Our results strongly suggest that one molecule of YcgR_LIC_11920_ binds a dimer of c-di-GMP, and SEC-MALS assays showed that the protein remains monomeric in the presence or absence of c-di-GMP, validating the structure-based predictions. Using the structure of YcgR_LIC_11920_ predicted by AlphaFold, we run molecular dynamics simulations to evaluate the effect of the GAZ domain on the c-di-GMP binding dynamics. These results showed that the PilZ–ligand interactions are stable, and the PilZN and PilZ domain interactions are mostly unaffected by the GAZ domain, although the simulations highlighted the presence of anticorrelations between PilZN and GAZ when binding to a c-di-GMP dimer.

Therefore, computational and biochemical evidence shows that the insertion of the GAZ domain did not affect LIC_11920s capacity to bind c-di-GMP. The capacity to bind c-di-GMP can also be expected for the other YcgR^GAZ^ homologues as all key binding motifs are strongly conserved in all members of the family. Still, determining whether the GAZ insertion changes c-di-GMP binding constants and affinity will require further experiments, and the functional significance of the GAZ domain in YcgR paralogs in *L. interrogans* remains unknown.

YcgR proteins are known to regulate flagellar torque, serving either as a clutch or as a brake in organisms such as *E. coli*, *B. subtilis*, and *V. vulnificus*. In these model organisms, the flagellar apparatus is composed of several subsystems, including the rotor (FliG) that interacts with the C-ring (FliM, FliN), the stator (MotA_5_MotB_3_), and the M-, S-, P-, and L-rings, the hook, and the extracellular filament. The cytoplasmic part of MotA interacts with FliG, a component of the C-ring, to power rotation of the flagellum. FliG polymerizes into a gear-like disk that sits below the flagellar basal body and is necessary for rotation. The number of FliG units may vary among species, but as protons pass through MotA_5_MotB_3_, MotA changes conformation and pushes on FliG to create torque in increments determined by the number of FliG subunits in the gear. MotA_5_MotB_3_ is the only known motor that uses energy from the transmembrane ion gradient instead of ATP to generate mechanical work.^61^ The interaction between the stator and rotor is dynamic. The motor is bidirectional: chemotactic signaling can cause a conformational change in the rotor, known as “switching”, which results in a change of the rotational direction of the motor. In *E. coli*, binding of c-di-GMP to the PilZ domain of YcgR allows the PilZ domain to interact with the MotA protein and the PilZN to bind to FliG, making YcgR act as a brake, or to modulate the torque of the flagellum.^26^ YcgR therefore works by disrupting the MotA–FliG interactions. Interestingly, the only YcgR protein described in the spirochete *Borrelia burgdorferi* (PlzA, codified by the locus_tag BB_BB_0733) binds c-di-GMP, and knockout of *plzA* reduced swarm diameter on plates, indicating its role in motility. In addition, Δ*plzA* mutants showed reduced infectivity in mice and lower survival in fed ticks compared to wild-type cells, highlighting the role of PlzA in the enzootic life cycle of this human pathogen.^62^

Although our data do not provide solid evidence for a direct interaction between YcgR^GAZ^ and FliG/MotA, the absence of the canonical YcgR in many Leptospirales and in *Thermospira aquatica* suggests at least one of the paralogs might still act as a flagellar torque regulator. The high degree of divergence among the paralogues, illustrated in Figure 1, suggests the (so far unknown) interaction surfaces between YcgR and FliG/MotA could have diverged significantly, but since most Leptospirales still preserve at least three copies of YcgR^GAZ^, these proteins probably still play important roles in these organisms.

We therefore propose that a hitherto number of unidentified members of the YcgR^GAZ^ subfamily from *L. interrogans*, when in complex with c-di-GMP, may also bind FliG and MotA at the cell poles, thus regulating the torque of the flagellum motors. Adding complexity to the system, *L. interrogans* harbors three copies of FliG (NCBI locus tags LIC_10023, LIC_11393, and LIC_11900) and two copies of MotA (NCBI locus tags LIC_12931 and LIC_10622). Notably, LIC_10622 is located just upstream of LIC_10623, one of the two MotB homologues in *Leptospira interrogans* (the other one being LIC_13339). This context mirrors the organization of the loci for known MotA and MotB orthologs and thus suggests that these genes will form the MotA_5_–MotB_3_ complex. To investigate the interaction between MotA_LIC_10622_ and FliGs, we utilized AlphaFold3 predictions to model the complex, and our results suggest the existence of interactions between these two flagellar components (Figures S10 and S11). Interestingly, a proteomic analysis of *L. interrogans* Copenhageni Fiocruz L1–130 resulted in the detection of at least 4 out of 6 YcgR^GAZ^ proteins within the cell at the same time as other components of the flagellar stator, such as MotA_LIC_10622_, MotB_LIC_10623_, and the three paralogs of FliG (Table S4). This evidence supports the idea that YcgR^GAZ^ might bind these proteins *in vivo* to regulate the flagellum rotation in spirochetes.

The evolutionary dynamics of the YcgR^GAZ^ subfamily in Leptospirales is dominated by duplications: in *L. interrogans*, ten different genes code for proteins that contain PilZ domains, and six of them are YcgR paralogues, all of which contain a GAZ insertion. Considering the roles of multiple copies of YcgR^GAZ^, we suggest two possible scenarios for the functions that members of this family might have acquired as they diverge from each other. In the first scenario, all copies of YcgR^GAZ^ preserve their interactions with flagellar proteins and thus continue to play a role in regulation of cell motility and shape similar to the previously described functions of their homologues in model organisms. Differences in gene expression patterns and binding affinities to either FliG or MotA could lead to a complex network of regulatory interactions between YcgR^GAZ^ and the flagellum as different members of the subfamily compete for the same binding sites. Alternatively, a second scenario would arise if only one of the YcgR^GAZ^ maintains its role as a flagellar regulator, while the other paralogs evolve new functions by adapting their interaction surfaces to bind other targets. At present, both scenarios are compatible with our experimental data, and the genomic context and evolutionary history of the family do not provide enough information to suggest which of the paralogs could still act as a flagellum regulator. Reality may correspond to a mix of these two simplified scenarios.

In conclusion, this study identified and characterized a novel subfamily of YcgR proteins, termed YcgR^GAZ^, which is uniquely expanded and diversified within the Leptospiraceae family, a group of spirochetes distinguished by their periplasmic flagellum with dual flagellar rotors located at each bacterial pole. These proteins retain the ability to bind c-di-GMP despite significant structural divergence, specifically due to the insertion of a GAF-like domain which we designate as the GAZ domain, within the PilZ domain—a novel feature observed for the first time in this study. The expansion and diversification of YcgR^GAZ^ in Leptospiraceae strongly suggests neofunctionalization, thus implying novel connections in *Leptospira*’s c-di-GMP signal transduction pathways governing motility and other physiological processes. The YcgR^GAZ^ protein family, therefore, could play a pivotal, yet unexplored, role in defining some of the unique features of spirochaetal and *Leptospira*’s biology.

## Methods

### Bioinformatic Analysis

#### Sequence Analysis

Iterative searches for homologues of YcgR^GAZ^ were run against a local copy of the nonredundant protein database, downloaded from the NCBI’s FTP site on 03/11/2023. Searches for PilZ and PilZN homologues were performed using all available Pfam models for these families and the software *hmmsearch* of the HMMER3 package, with an e-value cutoff of 10^–5^. Additional searches for PilZ homologues were performed using PSI-BLAST, for up to 3 iterations with an inclusion threshold e-value of 10^–2^. Additional searches for remote homologues were run on the HHpred and Phyre2 servers, against Hidden Markov models derived from the PDB, Pfam, and Scope databases. Further searches for structurally similar domains and proteins were run on the FoldSeek and Dali servers using the predicted structures for YcgR^GAZ^ homologues. Secondary and tertiary structures were extracted from PDB search results or predicted using the JPred server AlphaFold2 and ESMfold. Identification of conserved gene neighborhoods was performed using an *in-house* Python script that extracts genome annotation around target genes, followed by sequence clustering with MMseqs2 and protein domain annotation using HMMER3. Multiple sequence alignments were built using the L-INS-i algorithm implemented in the Mafft software, version v7.490, followed by manual curation. Phylogenetic inference was performed using the approximate maximum-likelihood method of FastTree, version 2.1.11. Unless stated otherwise, all software and online services mentioned above were run using default parameters.

#### Structure Prediction

All of the protein complexes were predicted using AlphaFold3, available in the AlphaFold Server, using the default parameters. Each sequence input generates five predicted models that were ranked by their respective *ranking_score*, and the highest scoring model was used.

### Molecular Docking and Molecular Dynamics

#### Ligand Preparation

The c-di-GMP molecule, represented by the SMILES code C1C2C(C(C(O2)N3C=NC4=C3N=C(NC4=O)N)O)OP(=O)(OCC5C(C(C(O5)N6C=NC7=C6N=C(NC7=O)N)O)OP(=O)(O1)O)O, was unprotonated (−2*e* charge) on the phosphate groups predicted to be charged at pH 7 with precision of 0.001, using the Dimorphite-DL program. This resulted in the unprotonated structure Nc1nc2c(ncn2C2OC3COP(=O)([O−])OC4C(COP(=O)([O−])OC3C2O)OC(n2cnc3c(=O)[n-]c(N)nc32)C4O)c(=O)[n-]1. The SMILES representation containing the protonation state at pH 7 was converted into a 3D structure using Chimera tools, which was then used for molecular docking. The code script of the Dimorphite-DL program was modified to obtain one SMILES representation, changing the count from 185 to 1. This structure was used in molecular docking simulation in order to obtain the YcgR_LIC_11920_/c-di-GMP monomer complex. The information on the protonated groups was included in the cocrystallized c-di-GMP molecule during construction of the YcgR_LIC_11920_/c-di-GMP dimer complex.

#### Molecular Docking and Molecular Dynamics

The 3D structure of YcgR_LIC_11920_ was predicted using AlphaFold2^63^ considering a minimized structure. The YcgR_LIC_11920_ structure model was prepared using the UCSF chimera tool for molecular docking simulations,^64^ according to references (65 and 66). Since dimeric c-di-GMP interacts with YcgR^21^ and also to be in agreement with our ITC experiments (2c-di-GMP: 1 × YcgR_LIC_11920_), we used cocrystallized c-di-GMP dimer (PDB 5Y6F(24)) in our molecular docking simulation. All histidines were protonated in the ε2 tautomer. To build a YcgR_LIC_11920_/c-di-GMP dimer complex, we restricted the atom positions of cocrystallized c-di-GMP dimer interacting with YcgR from *E. coli* (PDB 5Y6F(24)) and docked into the binding site. Subsequently, the c-di-GMP monomer (or dimer) was submitted to a systematic conformational assembly into the binding site using the autodocking Vina software.^67^ The grid was centered in the mean coordinate of the C_ζ_ atom from conserved arginines, the C_ζ_ atom of R112 and the C_ζ_ atom of R116. In the docking parameters, we used exhaustiveness and number of modes of 8 and 1, respectively. We differed in the molecule poses considering differences of 3 kcal/mol. The poses were evaluated by scoring representing the binding free energy. We performed nine consecutive minimizations of the YcgR_LIC_11920_/c-di-GMP dimer complex in maestro. YcgR_LIC_11920_/c-di-GMP complexes were submitted to molecular dynamics studies. All MD runs were performed in the Groningen machine for chemical simulation software (GROMACS, version 2022), using optimized potentials for liquid simulations for all-atom (OPLS-AA) force fields. Ligand topology was built in LigParGen web-based service, and the 1.14*CM1A charges were calculated considering charge −2 and 1 interaction. All systems were then explicitly solvated with TIP3P water models in a triclinic box and neutralized, keeping 150 mM NaCl concentration, and minimized until reaching a maximum force of 10.0 kJ mol^–1^ or a maximum number of steps of 5,000. The YcgR_LIC_11920_/c-di-GMP monomer was equilibrated consecutively in isothermal–isochoric (NVT) and isothermal–isobaric (1 bar; NpT) ensembles, both at temperature of 310 K for 1 ns (number of steps and intervals of 1,000,000 and 1 fs, respectively). All simulations were then performed in a periodic triclinic box, considering the minimum distance of 1.0 nm between any protein atom and triclinic box walls. Molecular dynamics runs were performed for 450 ns (number of steps and intervals of 450,000,000 and 1 fs, respectively) in NpT ensembles. The YcgR_LIC_11920_/c-di-GMP dimer was equilibrated consecutively in isothermal–isochoric (NVT) and isothermal–isobaric (1 bar; NpT) ensembles, both at temperature of 310 K for 2 ns (number of steps and intervals of 1,000,000 and 2 fs, respectively). All simulations were then performed in a periodic triclinic box considering the minimum distance of 1.0 nm between any protein atom and triclinic box walls. A molecular dynamics run was performed for 200 ns (number of steps and intervals of 100,000,000 and 2 fs, respectively) in NpT ensembles. We used the leapfrog algorithm to integrate Newton equations. We selected LINCS (LINear Constraint Solver) as a solver that satisfies holonomic constraints, whose number of iterations and order were 1 and 4, respectively. We used neighbor searching grid cells (cutoff scheme Verlet, frequency to update the neighbor list of 20 steps, and cutoff distance for the short-range neighbor list of 12 Å). In the van der Waals parameters, we smoothly switched the forces to zero between 10 and 12 Å. In electrostatic Coulomb, we used fast smooth Particle-Mesh Ewald (SPME) electrostatics for long-range electrostatics. In addition, we defined the distance for the Coulomb cutoff of 12 Å, interpolation order for PME for a value of 4 (cubic interpolation), and grid spacing for Fast Fourier Transform (FFT) of 1.6 Å. In temperature coupling, we used velocity rescaling with a stochastic term (V-rescale; modified Berendsen thermostat), and after obtaining two coupling groups (Protein/c-di-GMP dimer (or monomer) and water/ions), we considered them for time constant (0.1 ps) and 310 K as the reference temperature. In pressure coupling (NpT ensembles), we used a Parrinello–Rahman barostat (type isotropic, time constant of 1 ps, reference pressure of 1 bar and isothermal compressibility of 4.5 × 10^–5^ bar^–1^). In the molecular dynamics calculations, we used periodic boundary conditions in *xyz* coordinates (3D space). Next, we calculated root-mean-square deviation (RMSD) and root-mean-square fluctuation (RMSF) values using the GROMACS modules. We calculated enthalpy using MM-PBSA. Scripts to determine vectors and angles are available in the Supporting Information. The movies were performed in the UCSF chimera tool using the parameters of 10 steps.

### Cloning, Expression, and Purification of the YcgR_LIC_11920_ and Mutants

For the amplification of the gene of NCBI’s locus_tag LIC_11920, we used oligonucleotide sequences shown in Table S1. LIC_11920 was amplified from the *Leptospira interrogans* serovar Copenhageni Fiocruz L1–130 genome. Subsequently, both the PCR product and pET28a(+) plasmid were digested with the restriction enzymes *Nde*I and *Xho*I (FastDigest, Thermo Scientific). Following purification, the fragments were subjected to a ligation reaction using T4 DNA ligase (Thermo Scientific), with overnight incubation at 16 °C. The ligation reactions were used to transform chemically competent *E. coli* cells (Stellar and DH5α) through a heat shock protocol. Colonies that grow on kanamycin-containing plates were then inoculated into liquid LB medium, supplemented with the antibiotic, and incubated under agitation at 37 °C. Subsequent extraction of the plasmid DNAs was performed using the GeneJET miniprep kit (Thermo Scientific) with additional confirmatory digestions carried out and validated through electrophoresis on a 1% agarose gel. The same methodology was applied for the cloning of LIC_11920^R112A/R116A^, with double mutation in R112 and R116 to alanines.

YcgR_LIC_11920_ and YcgR_LIC_11920_^R112A/R116A^ were expressed in *E. coli* BL21-CodonPlus(DE3)-RIL cultures grown in 2xTY medium, supplemented with 50 μg/mL kanamycin and 15 μg/mL chloramphenicol at 37 °C until reaching an OD_600_ between 0.8 and 1.0. Induction was initiated with 0.5 mM isopropyl β-d-thiogalactopyranoside (IPTG) and continued for 4 h with agitation (180 rpm) at 37 °C. Cells were harvested by centrifugation (3500 × *g*, 15 min, 4 °C), resuspended in lysis buffer (50 mM Tris-HCl, 350 mM NaCl, 10% glycerol, pH 7.5, 0.03% Tween-20, 0.03% Triton X-100, and 100 μg mL^–1^ lysozyme), and lysed by sonication. Following centrifugation (35.000 × *g*, 40 min, 4 °C), the lysate was collected and loaded to a HisTrap-HP 5 mL column (GE Healthcare), washed using 20 CVs (Column Volumes) of buffer A (50 mM Tris-HCl, 350 mM NaCl, 20 mM imidazole, 10% glycerol, pH 7.5). Protein elution was performed using buffer B (50 mM Tris-HCl, 350 mM NaCl, 500 mM imidazole, 10% glycerol, pH 7.5) with an imidazole gradient from 0 to 100% B in 20 CVs. Fractions were analyzed via 15% SDS-PAGE, and proteins were concentrated using Amicon Ultracel-30K (Millipore), followed by size-exclusion chromatography (SEC) on a HiLoad 16/600 Superdex 200 pg column (GE Healthcare) equilibrated in SEC buffer (50 mM Tris-HCl, 350 mM NaCl, 10% glycerol, pH 7.5). Protein-containing fractions were pooled, once again concentrated using Amicon Ultracel-30K (Millipore), and stored at 4 °C.

### Multiangle Laser Light Scattering Coupled with Size-Exclusion Chromatography (SEC-MALS)

SEC-MALS analysis was employed to determine the molecular weight of YcgR_LIC_11920_. The protein was used at 42.8 μM, along with 400 μM c-di-GMP. For separation, protein samples (injection volume of 250–500 μL) traversed a Superdex 200 increase 10/300 pg column (GE Healthcare) coupled with a miniDAWN TREOS multiangle light scattering system and an Optilab rEX refractive index detector. The column was equilibrated with SEC buffer (50 mM Tris-HCl, 350 mM NaCl, 10% glycerol, pH 7.5), and c-di-GMP was added at a molar ratio of 1:2 protein with respect to the protein, according to a reference. Data analysis was executed using the Astra Software package, version 7.1 (Wyatt Technology Corp). Molecular weight calculations were based on the assumption of a refractive index increment (d*n*/d*c*) of 0.185 mL/g.

### Circular Dichroism (CD)

Circular dichroism (CD) spectroscopy was performed to analyze the secondary structure of the YcgR_LIC_11920_ in both the absence and presence of c-di-GMP and c-di-AMP. The protein was used at a concentration of 30 μM, while c-di-GMP was added at a final concentration of 150 μM. The CD measurements were conducted on a Jasco J-815 Spectrometer, with protein samples loaded into a 1 mm quartz cuvette. The spectra were acquired at 25 °C in the far-UV range of 190–260 nm, utilizing a 0.5 nm wavelength increment. The assays were performed using 1:2 purified protein (50 mM Tris-HCl, 50 mM NaCl, 10% glycerol, pH 7.5) and ligand.

### Isothermal Titration Calorimetry (ITC)

Isothermal titration calorimetry (ITC) was performed to assess the interaction between YcgR_LIC_11920_ and YcgR_LIC_11920(R112A/R116A)_ with c-di-GMP and c-di-AMP using a Malvern VP-ITC microcalorimeter at two different temperatures, 10 and 20 °C. The protein samples were diluted in an SEC buffer (50 mM Tris-HCl, 350 mM NaCl, 10% glycerol, pH 7.5) and loaded into the ITC cell, while ligands were also diluted in the same buffer and loaded into the syringe. The final concentration of protein used was 20 μM, and ligands were maintained at 400 μM. Injections of ten microliters of c-di-GMP solution were performed at 4 min intervals. The thermodynamic parameters of the binding reactions were fitted using the commercial Origin 7.0 program to derive Δ*H* (enthalpy change), *N* (stoichiometry), *K*_D_ (dissociation constant), and Δ*S* (entropy change) values. Commercially available lyophilized c-di-GMP (Cellco Biotech) was utilized, and for optimal monodispersity of c-di-GMP samples, solutions were incubated at 60 °C for 1 h before titration as suggested by Grzesiek and collaborators.^55^ The protein concentration was continuously monitored at 280 nm by using the theoretical protein extinction coefficient calculated through the online tool ExPASy ProtParam. A blank run corresponding to the titration of c-di-GMP into the SEC buffer was carried out to account for c-di-GMP dilution.

## Supporting Information Available

Ribbon representation showing structural changes in the YcgR_LIC_11920_/c-di-GMP dimer complex over a 200 ns simulation (MP4)
Ribbon representation illustrating structural changes at the binding site of YcgR_LIC_11920_ interacting with the c-di-GMP dimer over a 200 ns simulation (MP4)
Structures of proteins identified by PilZ models (XLSX)
Results of searches for GAZ domains, using iterative methods (XLSX)
Figures S1 to S10 and Tables S1 to S4 and the scripts script_extract_frames.py, script_vector.py, and script_angle.py (PDF)
