# Supplementary material for: Insertion of a Divergent GAF-like Domain Defines a Novel Family of YcgR Homologues That Bind c-di-GMP in Leptospirales
Source: ACS Omega. 2025 Jan 21;10(4):3988–4006. doi: 10.1021/acsomega.4c09917 (PMC11800159; doi:10.1021/acsomega.4c09917)
Supplement: Supplementary file 5 — ao4c09917_si_005.pdf [file ao4c09917_si_005.pdf]

Supporting Information

Insertion of a divergent GAF-like domain defines a novel family of YcgR homologs that binds c-di-GMP in *Leptospirales*

Aline Biazola Visnardi[1$], Rodolfo Alvarenga Ribeiro[1$], Anacleto Silva de Souza[1$], Tania Geraldine Churasacari Vinces[2], Edgar E. Llontop[3], Anielle Salviano de Almeida Ferrari[1], Pedro Antônio França Henrique[1], Daniela Valdivieso[1], Daniel Enrique Sánchez-Limache[1], Gabriela Roberto Silva[1], Eduardo Pereira Soares[1], Thomas Wittmann Cezar Santos[1,5], Chuck Shaker Farah[3], Rogerio Corte Sassonia[4], Roberto K. Salinas[3], Cristiane Rodrigues Guzzo[1*], Robson Francisco de Souza[1,5*]

[1] Department of Microbiology, Institute of Biomedical Sciences, University of São Paulo, São Paulo, Brazil.

[2] Department of Parasitology, Institute of Biomedical Sciences, University of São Paulo, Brazil.

[3] Department of Biochemistry, Institute of Chemistry, University of São Paulo, São Paulo, Brazil.

[4] Federal University of São Paulo, Department of Chemistry, São Paulo, Brazil.

[5] Graduate Program in Bioinformatics, University of São Paulo, São Paulo, Brazil.

[$]These authors contributed equally

*Corresponding author

E-mail: crisguzzo@usp.br and crisguzzo@gmail.com

E-mail: rfsouza@usp.br

To whom correspondence should be addressed:

Cristiane R. Guzzo, Ph.D, Department of Microbiology, Institute of Biomedical Sciences, University of São Paulo, Av. Prof. Lineu Prestes, 1374, Cidade Universitária, 05508-000, São Paulo/SP, Brazil, +55 11 3091-7298; E-mail: crisguzzo@usp.br

Robson Francisco de Souza, Ph.D, Department of Microbiology, Institute of Biomedical Sciences, University of São Paulo, Av. Prof. Lineu Prestes, 1374, Cidade Universitária, 05508-000, São Paulo/SP, Brazil, +55 11 3091-7298; E-mail: rfsouza@usp.br

Figure S1. 15% SDS-PAGE gel for testing the expression of full-length YcgRLIC_11920. 2
Figure S2. Determination of the molecular weight of YcgRLIC_11920 by SEC-MALS. 3
Figure S3. Thermal Stability Assessment of YcgRLIC_11920 in the Presence of Various Ligands 4
Figure S4. Replicate assays of ITC showing that YcgRLIC_11920 binds to c-di-GMP. 5
Figure S5. c-di-GMP, YcgRLIC_11920 and YcgRLIC_11920-cdi-GMP model structures. 5
Figure S6. Backbone root-mean-square deviation (RMSD) of the YcgRLIC_11920 interacting with monomer and dimer of c-di-GMP and radius of gyration (Rg) over time. 6
Figure S7. Distances between YcgRLIC_11920 and different chemical groups of c-di-GMP (in monomer and dimer forms). 7
Figure S8. Predicted structure of the protein from the locus DCF70_02805, a YcgRNpzN from the uncultivated Treponema UBA9738. 8
Figure S9. Predicted models of MotA5FliG3 using Alphafold3. 9
Table S1. Primers for the amplification of the wild-type LIC_11920 and mutant. 11
Table S2. Pearson correlation for the abundance of PilZ-like genes in Spirochaetes. 12
Table S3. Pearson correlation for the abundance of PilZ-like genes in all genomes. 12
Table S4. Estimated protein copies per cell for the detectable proteome in L. interrogans. 13
Execute in python "script_extract_frames.py" in directory containing the gromacs files 13
Execute in python "script_vector.py" in directory containing the gromacs files 14
Execute script_angle.py 14
Movie S1- Ribbon representation of structural changes of YcgRLIC_11920 /c-di-GMP dimer complex along 200 ns. 18
Movie S2- Ribbon representation of structural changes onto binding site of YcgRLIC_11920 interacting with c-di-GMP dimer along 200 ns. 18
Supplementary File S1 18
Supplementary File S2 18
Reference 19

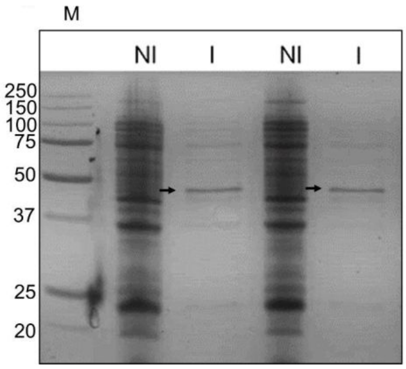

Figure S1. 15% SDS-PAGE gel for testing the expression of full-length YcgR$_{LIC\ 11920}$.
After obtaining clones encompassing the entire YcgR$_{LIC\ 11920}$ protein (1-389), designated as YcgR$_{LIC\ 11920(1-389)}$, expression tests were conducted in the *E. coli* BL21(DE3)-RIL strain at 37°C. Samples of non-induced (NI) and induced (I) *E. coli* BL21(DE3)-RIL cells. The total protein amount in the induced samples was lower compared to the non-induced samples. The samples were not normalized prior to loading. M corresponds to the Precision Plus Protein Standards unstained (BIO-RAD). The theoretical molecular weight of LIC_11920 is 46 kDa.

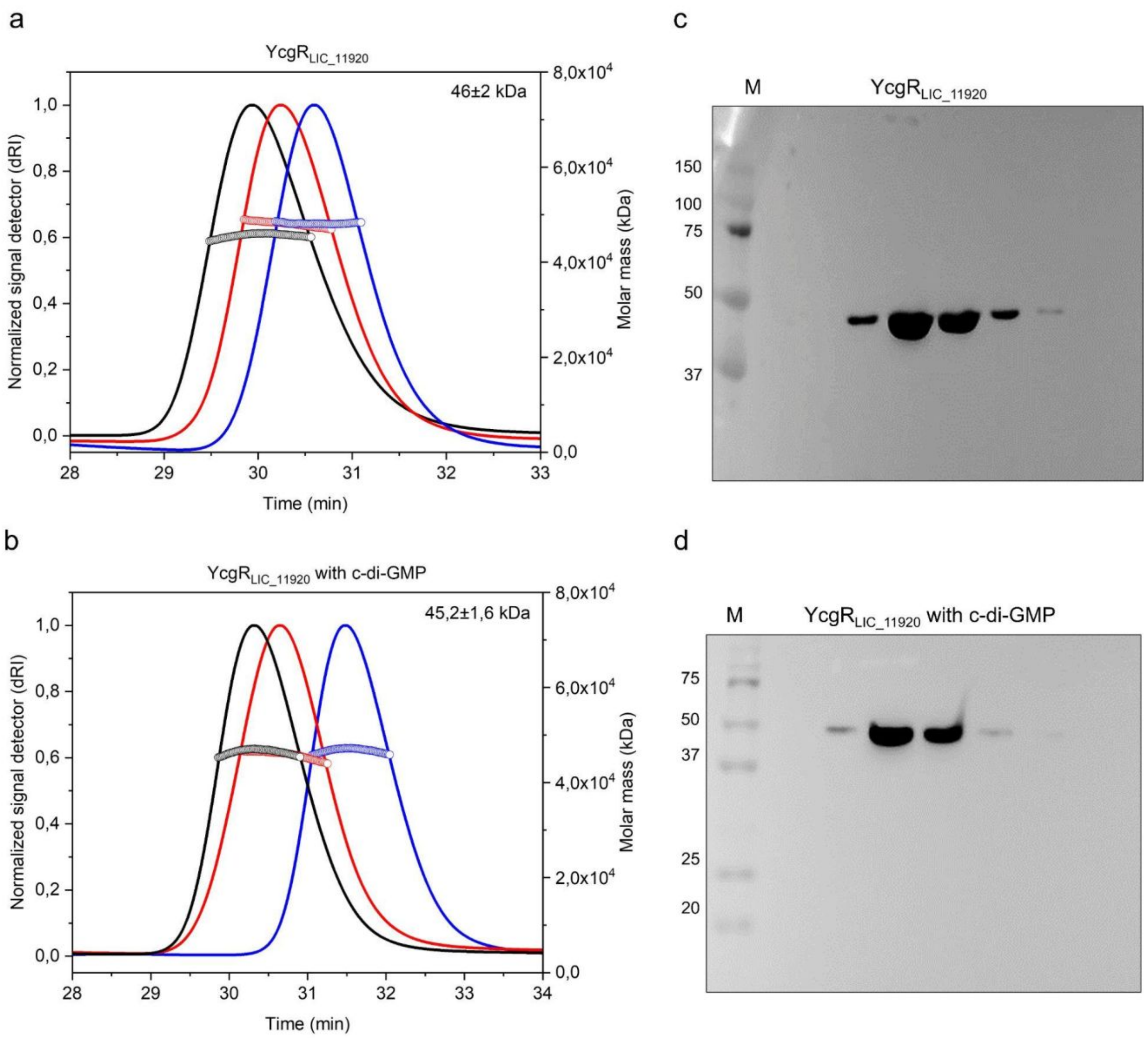

Figure S2. Determination of the molecular weight of YcgR$_{LIC_11920}$ by SEC-MALS.

Aliquots of 250 µL of YcgR$_{LIC_11920}$ (42 µM), in the absence (**a**) and presence (**b**) of c-di-GMP (400 µM), were applied into a Superdex200 Increase 10/300 pg column. Triplicates were performed and are shown in black, red, and blue. The lines represent the normalized refractive index (dRI) while the circles represent the molar mass distribution (kDa). YcgR$_{LIC_11920}$ eluted as a monomer, showing molecular weight of 46 and 45 kDa, in the absence and presence of c-di-GMP, respectively. Western blotting assays were performed to confirm the presence of YcgR$_{LIC_11920}$ in the SEC elution peak in the absence **(c)** and presence of c-di-GMP **(d)**. To confirm the monomeric state of YcgR$_{LIC_11920}$ in the presence and absence of c-di-GMP, triplicates of the same sample were analyzed and shown in different colors. The presence and absence of c-di-GMP does not affect the monomeric state of YcgR$_{LIC_11920}$.

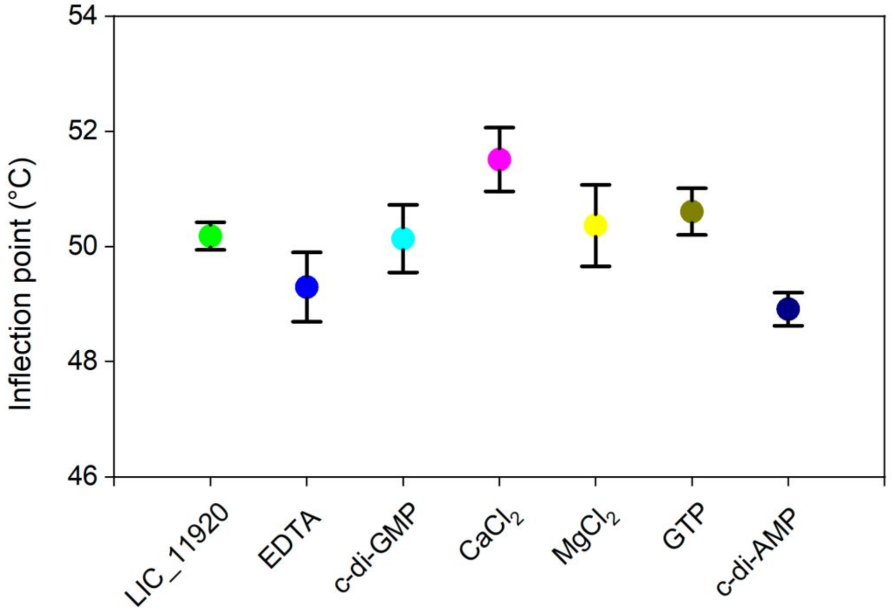

Figure S3. Thermal Stability Assessment of $\text{YcgR}_{\text{LIC\_11920}}$ in the Presence of Various Ligands

Thermal denaturation curves of $\text{YcgR}_{\text{LIC 11920}}$ were monitored using a circular dichroism (CD) spectrophotometer (JASCO) at 230 nm to evaluate its thermal stability in the presence of c-di-GMP and other ligands. No significant gain in thermal stability was observed with c-di-GMP. However, isothermal titration calorimetry (ITC) experiments confirmed the interaction between $\text{YcgR}_{\text{LIC 11920}}$ and c-di-GMP. Additionally, the presence of c-di-AMP and GTP did not enhance the thermal stability of the protein. In contrast, a slight increase in the melting temperature (TM) of approximately 1°C was detected in the presence of calcium chloride ($CaCl_2$), indicating a potential calcium-binding capability. No improvement in thermal stability was observed with magnesium chloride ($MgCl_2$).

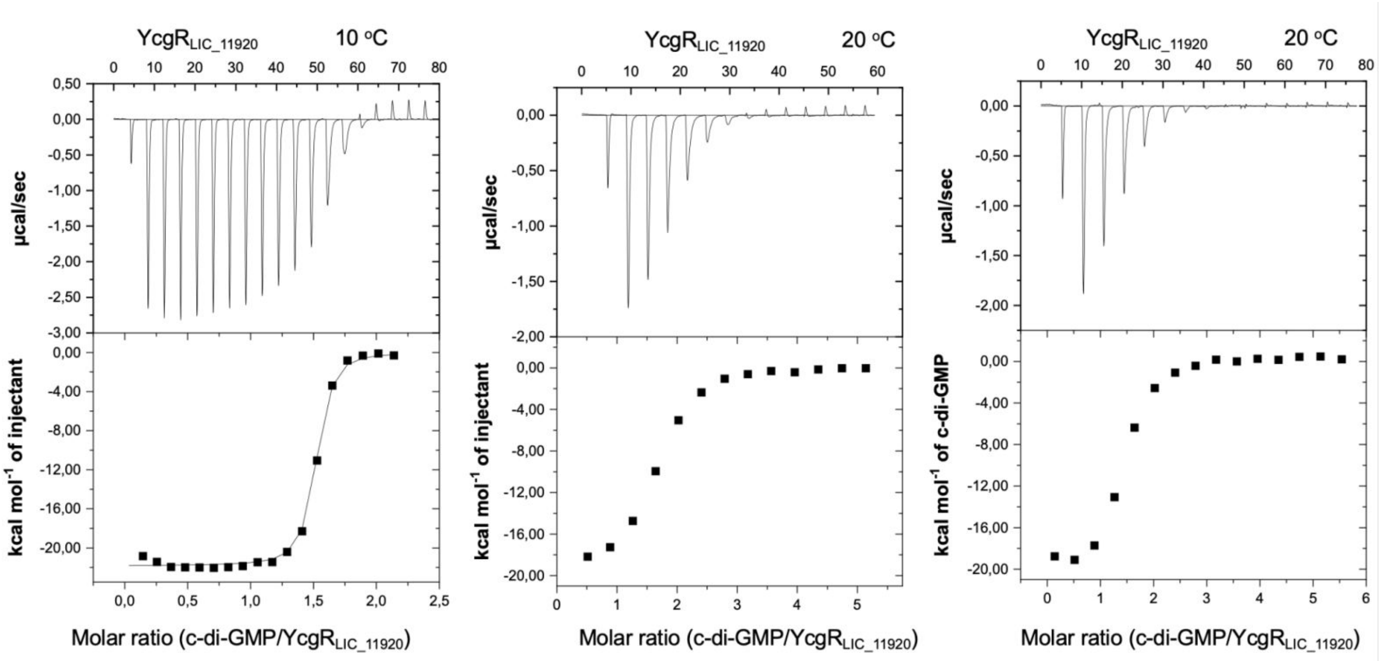

Figure S4. Replicate assays of ITC showing that YcgR$_{LIC_11920}$ binds to c-di-GMP. Exothermic profile of interaction between YcgR$_{LIC_11920}$ and c-di-GMP at 10°C and at 20°C.

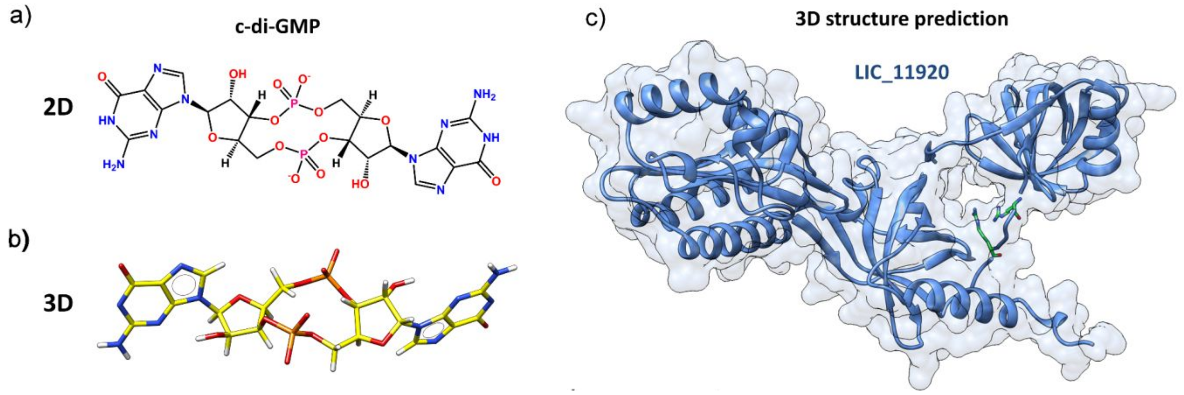

Figure S5. c-di-GMP, YcgR$_{LIC_11920}$ and YcgR$_{LIC_11920}$-cdi-GMP model structures. **a**) 2D structural representation of c-di-GMP with a total charge of -2e. The phosphate groups are predicted to be charged at pH 7 with precision 0.001. **b**) The 2D representation was converted into a 3D structure using Chimera tools. **c**) The 3D model of relaxed YcgR$_{LIC_11920}$ predicted by Alphafold.

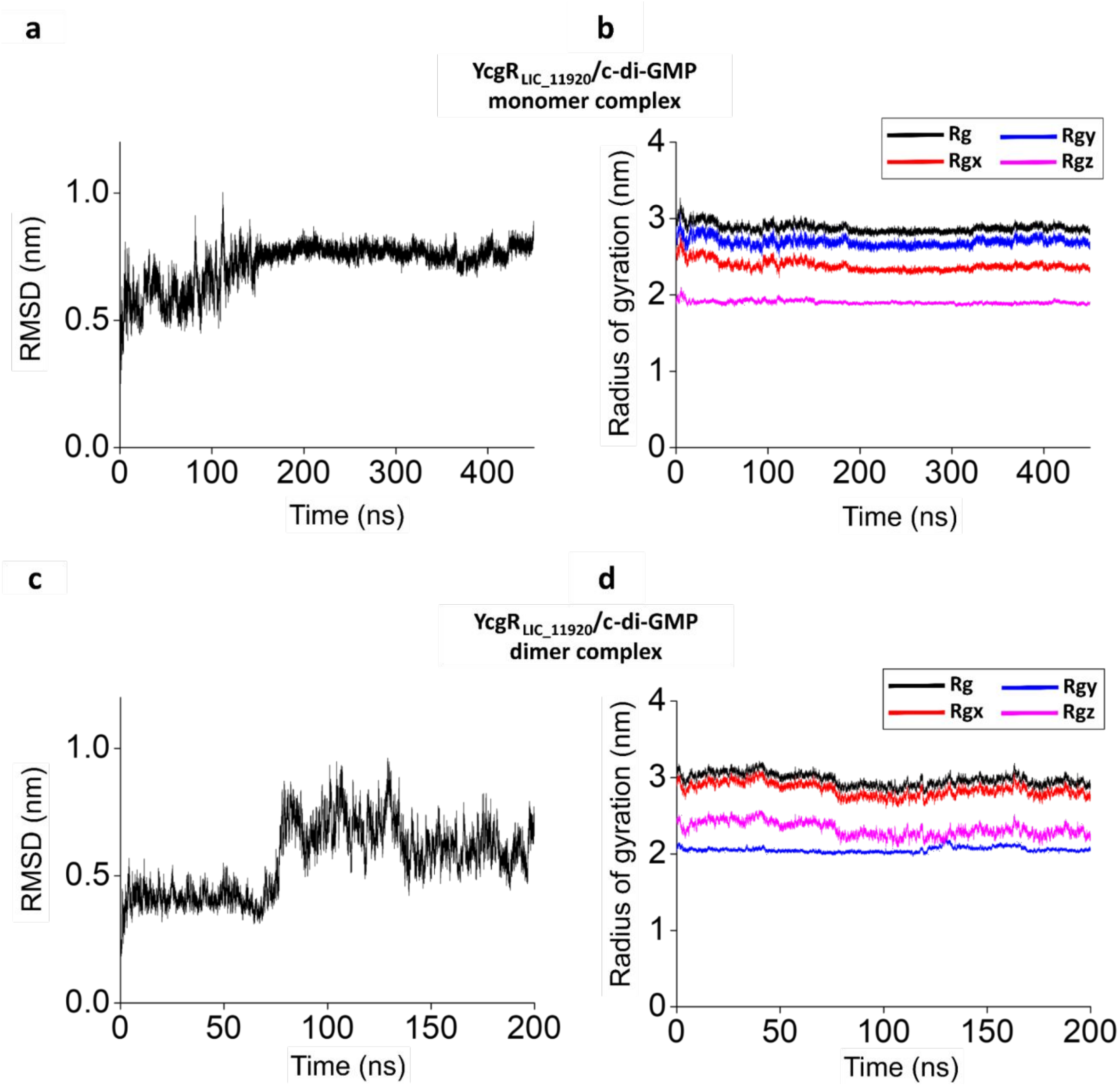

<u>Figure S6.</u> Backbone root-mean-square deviation (RMSD) of the YcgR$_{LIC_11920}$ interacting with monomer and dimer of c-di-GMP and radius of gyration (Rg) over time.

a) Root-mean-square deviation (RMSD) of the YcgR$_{LIC_11920}$ backbone over time. **b**) The radius of gyration (Rg) of the YcgR backbone over time, with Rgx, Rgy, and Rgz representing the components along the x, y, and z axes, respectively. **c**) Root-mean-square deviation (RMSD) of the YcgR$_{LIC_11920}$ backbone over time. **d**) The radius of gyration (Rg) of the YcgR backbone over time, with Rgx, Rgy, and Rgz representing the components along the x, y, and z axes, respectively. Panels **a)** and **b)** were built considering the simulation of YcgR$_{LIC_11920}$ interacting with c-di-GMP monomer while **c)** and **d)** were constructed considering the interaction between YcgR$_{LIC_11920}$ and c-di-GMP dimer. Notably, in all cases, both Rg and RMSD converged.

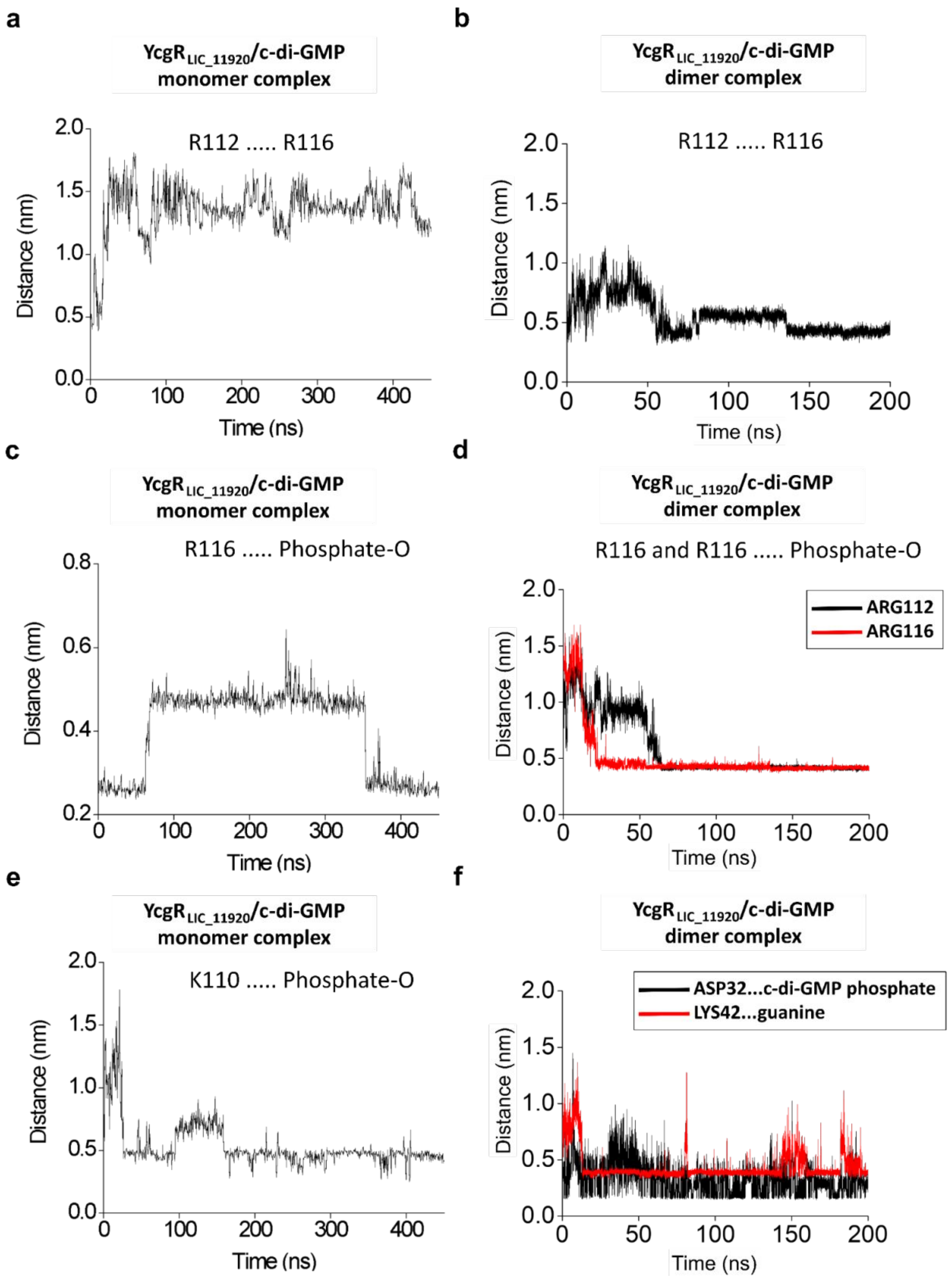

Figure S7. Distances between YcgR$_{LIC_11920}$ and different chemical groups of c-di-GMP (in monomer and dimer forms).

a) Distance between R112 and R116 of the YcgR$_{LIC_11920}$ during the interaction with c-di-GMP monomer. **b)** Distance between R112 and R116 of the YcgR$_{LIC_11920}$ during the interaction with c-di-GMP dimer. **c)** Distance between R116 and phosphate group of the c-di-GMP monomer. **d)** Distance between R112 and oxygen from the phosphate group and between R116 and oxygen from the phosphate group from one of the molecules that constitute the c-di-GMP dimer. **e)** Distance between K110 and phosphate group of the c-di-GMP monomer. **f)** Distance between D32 and phosphate group and between K32 and guanine group from one of the molecules that constitute the c-di-GMP dimer.

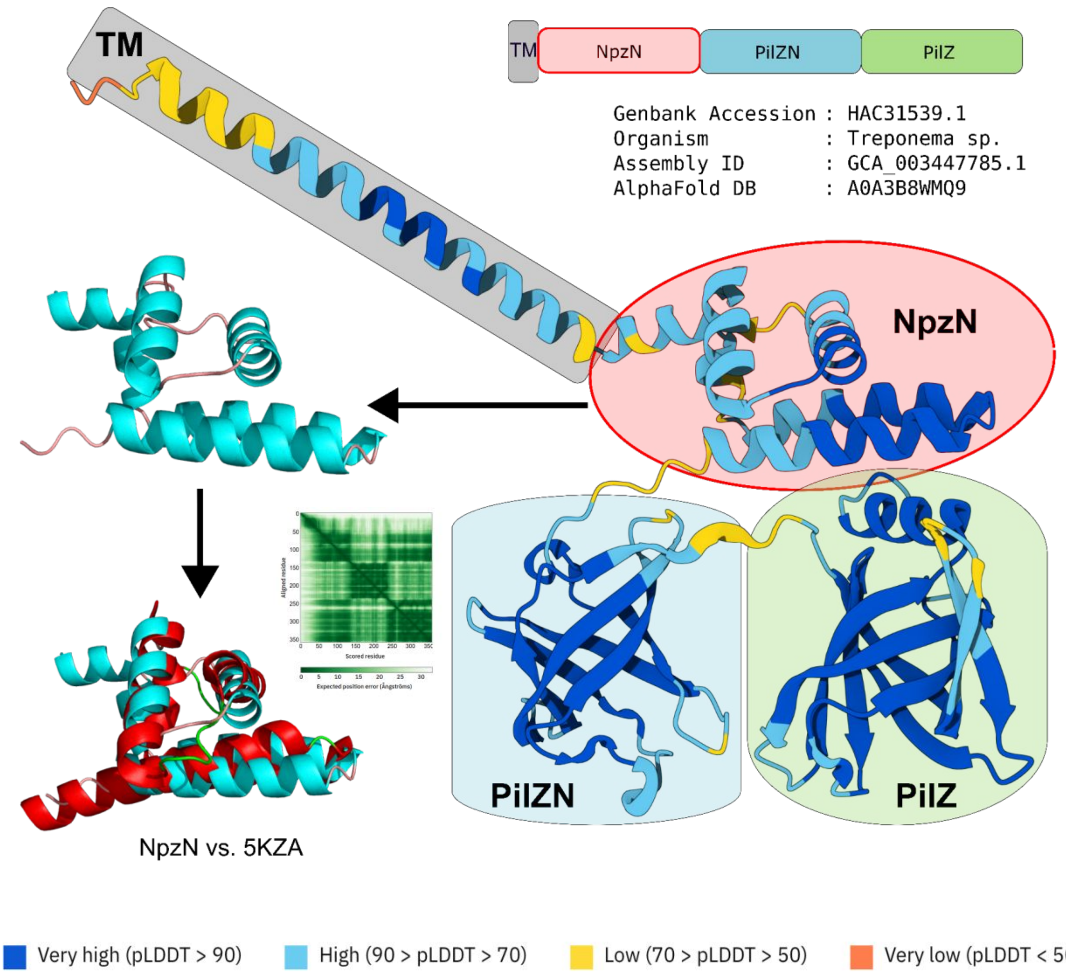

Figure S8. Predicted structure of the protein from the locus DCF70_02805, a YcgR^{NpzN} from the uncultivated *Treponema* UBA9738.

This genome was recovered from the metagenomic sample SRX290711. The predicted structure was obtained from DeepMind's AlphaFold Database. The color gradient of the structure corresponds to the predicted local distance difference test (pLDDT) confidence values. A simplified linear representation of the protein domain architecture is shown in the upper right corner. Lower left: structural superposition of the NpzN domain and the viral matrix protein (PDB: 5KZA, Dali Z-score: 6.0, Dali RMSD: 3.0, FoldSeek TM-score: 0.5, FoldSeek Probability: 14%) was built using the CE-align method implemented in PyMol. At the center, the green gradient plot shows the predicted aligned error (PAE) score from AlphaFold. Low values of PAE for residues in NpzN and the helix in PilZ suggests these residues could make contacts that stabilize NpzN's position. The Raus Sarcoma retrovirus matrix protein has affinity for regions of the cellular membrane of humans that are enriched in and similar activity is also possible for NpzN and the bacterial membrane. Membrane insertion mediated by the N-terminal transmembrane helix could help target YcgR^{NpzN} to the flagellar complex.

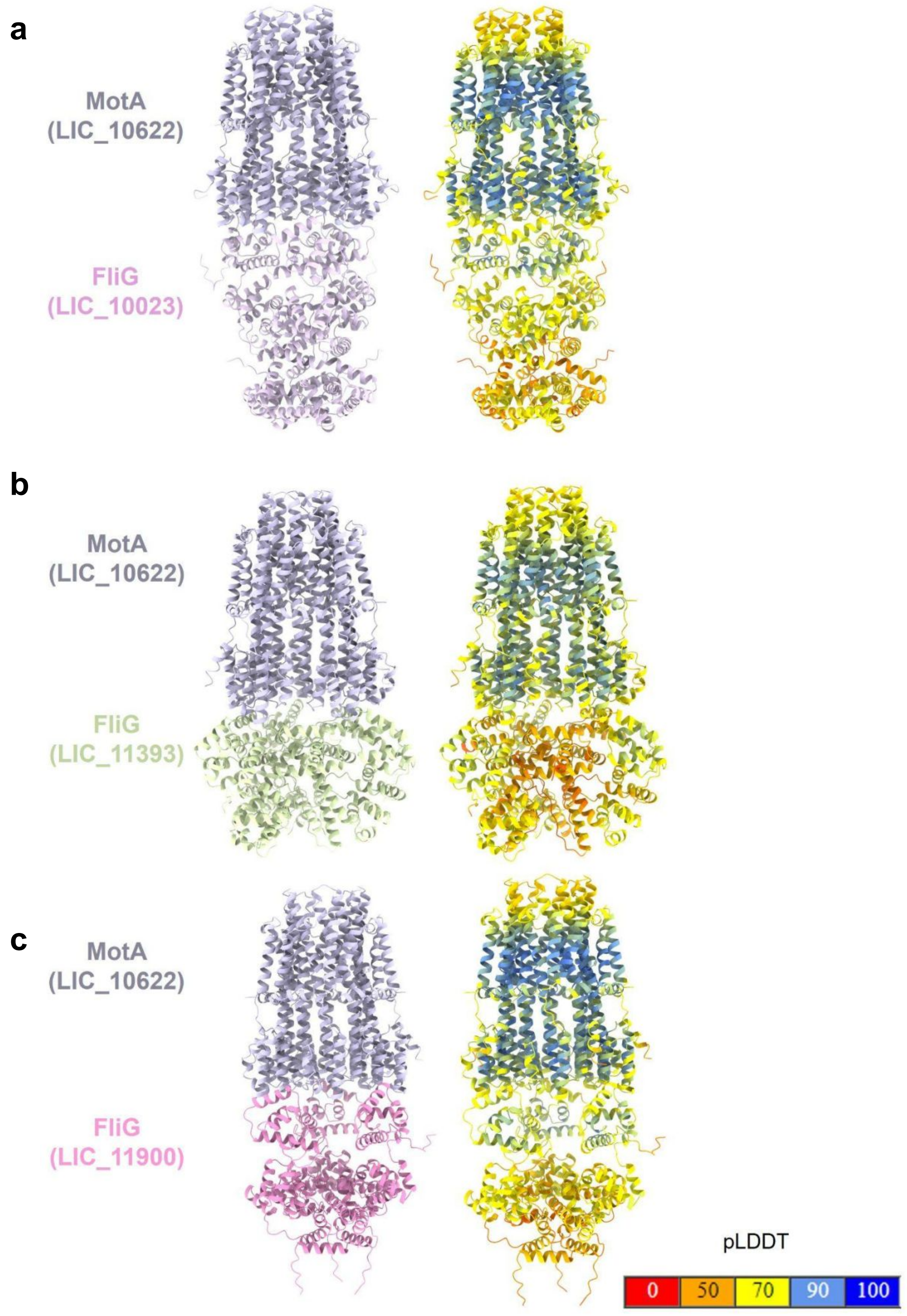

Figure S9. Predicted models of $MotA_5FliG_3$ using Alphafold3.

Predicted models of the complex between MotA and the three FliG paralogs ($MotA_5FliG_3$) colored by pLDDT (Predicted Local Distance Difference Test). The locus_tag of each protein is shown. **(a)** LIC_10622 (MotA) and LIC_10023 (FliG) complex model; **(b)** LIC_10622 (MotA) and LIC_11393 (FliG) complex model; **(c)** LIC_10622 (MotA) and LIC_11900 (FliG) complex model. We also tried to predict the

structure of these complexes bound to $YcgR_{LIC\_11920}$, but we did not obtain good predictions (data not shown). We believe that our difficulty in predicting the structure of $YcgR_{LIC\_11920}$ bound to the complex is due to the inability to add the c-di-GMP ligand, which modulates the activity of this protein and allows the YcgR to bind MotA and FliG.

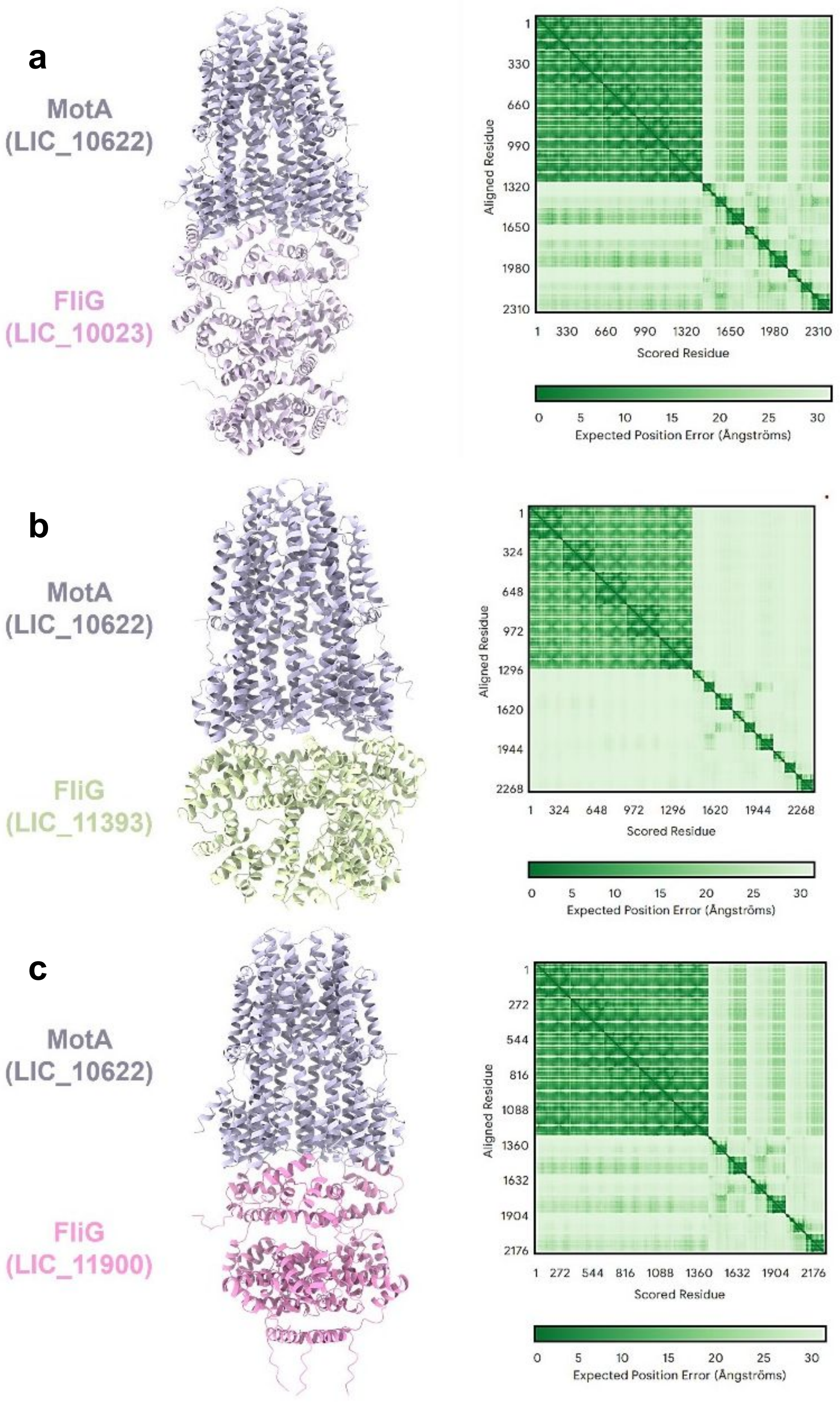

Figure S10. Predicted models using AlphaFold3[1] of the complex between MotA and the three FliG paralogs ($MotA_5FliG_3$): (a) LIC_10622 (MotA) and LIC_10023 (FliG) complex model; **(b)** LIC_10622 (MotA) and LIC_11393 (FliG) complex model;

(c) LIC_10622 (MotA) and LIC_11900 (FliG) complex model. We also tried to predict the structure of these complexes bound to $YcgR_{LIC\_11920}$, but we did not obtain good predictions (data not shown). We believe that our difficulty in predicting the structure of $YcgR_{LIC\_11920}$ bound to the complex is due to the inability to add the c-di-GMP ligand, which modulates the activity of this protein and allows the YcgR to bind MotA and FliG. The confidence metrics of each predicted structure are available.

Table S1. Primers for the amplification of the wild-type LIC_11920 and mutant. Three distinct constructions were designed. LIC_11920_1F: Forward primer for amplifying from the 1st position of the LIC_11920 gene. LIC_11920_15F: Forward primer for amplifying from the 15th position of the LIC_11920 gene. LIC_11920_109F: Forward primer for amplifying from the 109th position of the LIC_11920 gene. LIC_11920_389R: Reverse primer for amplifying from the 389th position of the LIC_11920 gene. LIC_11920_R112A_R116A_F: Forward primer designed for introducing specific point mutations (R112A and R116A) from LIC_11920-pET28a(+) product. LIC_11920_R112A_R116A_R: Reverse primer designed for introducing specific point mutations (R112A and R116A) in the LIC_11920 gene. A singular mutant construct was built with the oligonucleotide sequences utilized for plasmid constructions shown for constructs LIC_11920_R112A_R116A_F and LIC_11920_R112A_R116A_R. LIC_11920 was amplified from Leptospira interrogans serovar Copenhageni Fiocruz L1-130. The constructions LIC_11920_15F (forward, starting at 15 position) and LIC_11920_109F (forward, starting at 109 position) although the amplifications were a success, we didn't go ahead with the expressions and purification.

Construct	Restriction Enzymes	Sequences Primers 5'-3'	Tm (°C)
LIC_11920_1F	*Nde*I	AAAACATATGGAAACAATTCAGAGAAAAAA	59
LIC_11920_389R	*Xho*I	AAAACTCGAGTCAAAATTGGCTTTGAATTGG	59
LIC_11920_R112A_R116A_F	*Nde*I	CGATCGCGAGCAAAGAAGCAAGTCTCAACGCAATCGTACCTCCAGAAGG	59
LIC_11920_R112A_R116A_R	*Xho*I	CCTTCTGGAGGTACGATTGCGTTGAGACTTGCTTCTTTGCTCGCGATCG	59

Table S2. Pearson correlation for the abundance of PilZ-like genes in Spirochaetes.

The number of genes per genome was used as input to the Pandas *corr()* method, using default parameters. Genomes from RefSeq were preferred over their Genbank counterparts. The PilZN subfamily refers to proteins containing instances of PilZ-like domains that were not recognized by Pfam models for the PilZ family and didn't have the known c-di-GMP binding motifs of the PilZ family.

Pearson correlation coefficient (Spirochaetes)				
	**YcgR**	**$YcgR^{NpzN}$**	**PilZN**	**PilZ**
**$YcgR^{GAZ}$**	-0.757803	-0.215246	-0.083370	0.797714
**YcgR**		0.310424	0.138760	-0.686317
**NpzN**			0.229131	-0.118134
**PilZN**				-0.041639

Table S3. Pearson correlation for the abundance of PilZ-like genes in all genomes.

The number of genes per genome was used as input to the Pandas *corr()* method, using default parameters. Genomes from RefSeq were preferred over their Genbank counterparts. The PilZN subfamily refers to proteins containing instances of PilZ-like domains that were not recognized by Pfam models for the PilZ family and didn't have the known c-di-GMP binding motifs of the PilZ family.

Pearson correlation coefficient (all genomes)				
	**YcgR**	**$YcgR^{NpzN}$**	**PilZN**	**PilZ**
**$YcgR^{GAZ}$**	-0.077811	0.005993	-0.002261	0.030230
**YcgR**		0.006146	-0.033958	-0.050374
**NpzN**			0.043059	-0.017648
**PilZN**				-0.014971

Table S4. Estimated protein copies per cell for the detectable proteome in *L. interrogans.*
Data from a proteome of the cell sample subjected to extensive mapping via LC-MS/MS experiments [2].

Accession number	Locus_tag	Protein description	Copies/cell spectral counts
AAS71482.1	LIC_12931	MotA	NI
AAS69243.1	LIC_10622	MotA	200
AAS69244.1	LIC_10623	MotB	250
YP_003244.1	LIC_13339	MotB	NI
AAS68660.1<br>YP_000023.1	LIC_10023	FliG	11
AAS69993.1	LIC_11393	FliG	47
AAS70485.1	LIC_11900	FliG	24
YP_001866.1	LIC_11920	$YcgR^{GAZ}$	132
YP_002643.1	LIC_12723	$YcgR^{GAZ}$	NI
YP_002474.1	LIC_12546	$YcgR^{GAZ}$	37
YP_002907.1	LIC_12994	$YcgR^{GAZ}$	249
YP_000049.1	LIC_10049	$YcgR^{GAZ}$	24
WP_000357129	LIC_RS11585<br>(LIC14002)	$YcgR^{GAZ}$	?

NI. Not identified
? We could not identify the protein in the list table of the article [2]

Execute in python "script_extract_frames.py" in directory containing the gromacs files

```
import subprocess

def extract_frames():
        total_time_ns = 200  # Total time in nanoseconds
        time_interval_ns = 0.5  # Time interval for extracting frames
in nanoseconds

        num_frames = int(total_time_ns / time_interval_ns)

        for i in range(num_frames):
            time_ns = i * time_interval_ns
            command = f'echo 1 | gmx trjconv -s ../md.tpr -f
../md_center_fit.xtc -n ../index.ndx -tu ns -o frame_{time_ns}.pdb -
dump {time_ns}'
            subprocess.run(command, shell=True)

if __name__ == "__main__":
    extract_frames()
```

Execute in python "script_vector.py" in directory containing the gromacs files

```
import os
import numpy as np
import MDAnalysis as mda

def calculate_center_of_mass(frames_dir, domain, output_file):
    with open(output_file, 'w') as f:
        f.write("time,xc,yc,zc\n")
        for frame_file in sorted(os.listdir(frames_dir)):
            if frame_file.endswith('.pdb'):
                frame_path = os.path.join(frames_dir, frame_file)
                u = mda.Universe(frame_path)
                residues = u.select_atoms(domain)
                center_of_mass = residues.center_of_mass()
                differences = residues.positions - center_of_mass
                resultant_vector = np.sum(differences, axis=0)
                time = float(frame_file.split('_')[1].split('.pdb')[0])

f.write(f"{time},{resultant_vector[0]},{resultant_vector[1]},{resultant
_vector[2]}\n")

frames_dir = "../frames_steps_0.5ns"  # Frames directory
domains = {
    "pilz": "resid 1:12 or resid 110:149 or resid 288:389",
    "gaz": "resid 150:287",
    "pilzn": "resid 13:109"
}

for name, selection in domains.items():
    calculate_center_of_mass(frames_dir, selection,
f"cm_relative_{name}.csv")
```

Execute script_angle.py

```
import os

import numpy as np

import MDAnalysis as mda

from itertools import combinations

import csv



def calculate_center_of_mass(frames_dir, domain, output_file):

    with open(output_file, 'w', newline='') as f:
```

```
        writer = csv.writer(f)

        writer.writerow(["time", "xc", "yc", "zc"])


        for frame_file in sorted(os.listdir(frames_dir)):

            if frame_file.endswith('.pdb'):

                frame_path = os.path.join(frames_dir, frame_file)

                u = mda.Universe(frame_path)

                residues = u.select_atoms(domain)

                center_of_mass = residues.center_of_mass()

                differences = residues.positions - center_of_mass

                resultant_vector = np.sum(differences, axis=0)

                time = float(frame_file.split('_')[1].split('.pdb')[0])

                writer.writerow([time, resultant_vector[0],
resultant_vector[1], resultant_vector[2]])


frames_dir = "../frames_steps_0.5ns"  # Frames directory


domains = {

    "pilz": "resid 1:12 or resid 110:149 or resid 288:389",

    "gaz": "resid 150:287",

    "pilzn": "resid 13:109"

}


# Calculate the resultant vectors for each domain


results = {}


for name, selection in domains.items():
```

```
    output_file = f"resulting_vector_{name}.csv"

    calculate_center_of_mass(frames_dir, selection, output_file)

    results[name] = output_file


# Calculate the angles between the resultant vectors of each domain
combination


combinations_list = list(combinations(domains.keys(), 2))

for comb in combinations_list:

    combination_name = f"angle_{comb[0]}_vs_{comb[1]}"

    output_file = f"{combination_name}.csv"


    with open(output_file, 'w', newline='') as f:

        writer = csv.writer(f)

        writer.writerow(["time", "angle"])


        with open(results[comb[0]], 'r') as f1, open(results[comb[1]],
'r') as f2:

            reader1 = csv.reader(f1)

            reader2 = csv.reader(f2)

            next(reader1)  # Skip header

            next(reader2)  # Skip header


            for row1, row2 in zip(reader1, reader2):

                time1, vector1 = float(row1[0]), np.array(row1[1:],
dtype=float)

                time2, vector2 = float(row2[0]), np.array(row2[1:],
dtype=float)
```

```
                # Calculate angle between vectors

                cos_theta = np.dot(vector1, vector2) /
(np.linalg.norm(vector1) * np.linalg.norm(vector2))

                angle = np.arccos(np.clip(cos_theta, -1, 1)) * 180 /
np.pi

                writer.writerow([time1, angle])


print("Angle calculation completed!")
```

Movie S1- Ribbon representation of structural changes of $YcgR_{LIC\_11920}$ /c-di-GMP dimer complex along 200 ns.

Movie shows the conformational changes of $YcgR_{LIC\_11920}$/c-di-GMP dimer complex for all MD trajectories. The $YcgR_{LIC\_11920}$ and c-di-GMP are colored in blue and yellow, respectively. The movies were performed in the UCSF chimera tool using the parameters of 10 steps.

Movie S2- Ribbon representation of structural changes onto binding site of $YcgR_{LIC\_11920}$ interacting with c-di-GMP dimer along 200 ns.

Movie shows the conformational changes onto the binding site of $YcgR_{LIC\_11920}$ interacting with c-di-GMP dimer for all MD trajectories. The $YcgR_{LIC\_11920}$ and c-di-GMP are colored in blue and yellow, respectively. The movies were performed in the UCSF chimera tool using the parameters of 10 steps

Supplementary File S1

Supplementary File S2

Reference

(1) Abramson, J.; Adler, J.; Dunger, J.; Evans, R.; Green, T.; Pritzel, A.; Ronneberger, O.; Willmore, L.; Ballard, A. J.; Bambrick, J.; Bodenstein, S. W.; Evans, D. A.; Hung, C.-C.; O'Neill, M.; Reiman, D.; Tunyasuvunakool, K.; Wu, Z.; Žemgulytė, A.; Arvaniti, E.; Beattie, C.; Bertolli, O.; Bridgland, A.; Cherepanov, A.; Congreve, M.; Cowen-Rivers, A. I.; Cowie, A.; Figurnov, M.; Fuchs, F. B.; Gladman, H.; Jain, R.; Khan, Y. A.; Low, C. M. R.; Perlin, K.; Potapenko, A.; Savy, P.; Singh, S.; Stecula, A.; Thillaisundaram, A.; Tong, C.; Yakneen, S.; Zhong, E. D.; Zielinski, M.; Žídek, A.; Bapst, V.; Kohli, P.; Jaderberg, M.; Hassabis, D.; Jumper, J. M. Accurate Structure Prediction of Biomolecular Interactions with AlphaFold 3. *Nature* **2024**, 1–3. https://doi.org/10.1038/s41586-024-07487-w.

(2) Malmström, J.; Beck, M.; Schmidt, A.; Lange, V.; Deutsch, E. W.; Aebersold, R. Proteome-Wide Cellular Protein Concentrations of the Human Pathogen Leptospira Interrogans. *Nature* **2009**, *460* (7256), 762–765. https://doi.org/10.1038/nature08184.
